# Analysis of the cancer genome atlas (TCGA) database identifies an inverse relationship between interleukin-13 receptor α1 and α2 gene expression and poor prognosis and drug resistance in subjects with glioblastoma multiforme

**DOI:** 10.1007/s11060-017-2680-9

**Published:** 2017-11-22

**Authors:** Jing Han, Raj K. Puri

**Affiliations:** Tumor Vaccines and Biotechnology Branch, Division of Cellular and Gene Therapies, Office of Tissues and Advanced Therapies, Center for Biologics Evaluation and Research, Food and Drug Administration, WO Bldg. 71, Rm 5342, CBER/FDA, 10903 New Hampshire Ave., Silver Spring, MD 20993 USA

**Keywords:** GBM (glioblastoma multiforme), TCGA (the cancer genome atlas), *IL-13Rα1*, *IL-13Rα2*, Biomarker

## Abstract

Glioblastoma multiforme (GBM) is the most common primary brain tumor in adults. A variety of targeted agents are being tested in the clinic including cancer vaccines, immunotoxins, antibodies and T cell immunotherapy for GBM. We have previously reported that IL-13 receptor subunits α1 and α2 of IL-13R complex are overexpressed in GBM. We are investigating the significance of *IL-13Rα1* and *α2* expression in GBM tumors. In order to elucidate a possible relationship between *IL-13Rα1* and *α2* expression with severity and prognoses of subjects with GBM, we analyzed gene expression (by microarray) and clinical data available at the public The Cancer Genome Atlas (TCGA) database (Currently known as Global Data Commons). More than 40% of GBM samples were highly positive for *IL-13Rα2* mRNA (Log2 ≥ 2) while only less than 16% samples were highly positive for *IL-13Rα1* mRNA. Subjects with high *IL-13Rα1* and *α2* mRNA expressing tumors were associated with a significantly lower survival rate irrespective of their treatment compared to subjects with *IL-13Rα1* and *α2* mRNA negative tumors. We further observed that *IL-13Rα2* gene expression is associated with GBM resistance to temozolomide (TMZ) chemotherapy. The expression of *IL-13Rα2* gene did not seem to correlate with the expression of genes for other chains involved in the formation of IL-13R complex (*IL-13Rα1* or *IL-4Rα*) in GBM. However, a positive correlation was observed between *IL-4Rα* and *IL-13Rα1* gene expression. The microarray data of *IL-13Rα2* gene expression was verified by RNA-Seq data. In depth analysis of TCGA data revealed that immunosuppressive genes (such as *FMOD, CCL2, OSM*, etc.) were highly expressed in *IL-13Rα2* positive tumors, but not in *IL-13Rα2* negative tumors. These results indicate a direct correlation between high level of *IL-13R* mRNA expression and poor patient prognosis and that immunosuppressive genes associated with *IL-13Rα2* may play a role in tumor progression. These findings have important implications in understanding the role of IL-13R in the pathogenesis of GBM and potentially other cancers.

**Electronic supplementary material:**

The online version of this article (10.1007/s11060-017-2680-9) contains supplementary material, which is available to authorized users.

## Introduction

Glioblastoma multiforme (GBM) is a devastating brain tumor with extremely poor prognosis because of its diffusive and infiltrative nature, which is marked cytological heterogeneity. It is one of the most aggressive and common malignant brain tumors accounting for more than 50% of all gliomas [1]. GBM tumors are histologically and molecularly diverse, exhibiting heterogeneity both between patients and within individual tumors [2]. Several distinct GBM subtypes have been identified based on the gene expression-based molecular classification. Three glioblastoma subtypes were defined based on patient prognosis and gene expression clustering; proneural, proliferative, and mesenchymal subtypes [3]. Subsequently, gene expression studies from The Cancer Genome Atlas (TCGA) dataset defined four distinct glioblastoma subclasses, including proneural, neural, classical, and mesenchymal [4]. Kim et al. classified GBMs into three prognostic groups, which were different from the previously identified subtypes, and identified a 42 probe set of gene signatures, which associated with tumor aggressiveness and related with the epithelial-mesenchymal transition (EMT) process [5].

Currently, the standard treatment for GBM includes surgical resection followed by radiation therapy, chemotherapy, or a combination of these therapies. Despite the use of these therapies, the median survival time in patients remains dismal, at approximately 1 year. GBM remains an incurable disease with few therapeutic advances over the past several decades. Novel approaches are under development targeting glioma cell surface antigens and receptors, tumor invasion, angiogenesis, proliferation, immune escape, and tumor recurrence [6]. A relatively new form of therapy involves tumor targeting, which relies on the identification of unique or over-expressed cell surface receptors or antigens on tumor cells. One of the extensively studied cell-surface targets is interleukin-13 receptor alpha-2 chain (IL-13Rα2). This receptor is one of the two chains of the IL-13R complex [7]. IL-13Rα2 binds IL-13 with high affinity and has received significant attention in brain tumor therapy because it is overexpressed by high-grade glioblastomas, but not expressed at significant levels by normal brain tissue [6–8]. In addition, it has been shown that IL-13Rα2 promotes tumor invasion and metastasis in mouse models of human pancreatic and ovarian cancers [9, 10]. It is also shown that IL-13Rα2 can protect tumor cells from apoptosis thereby contributing to tumor growth [11]. The IL-13Rα2 chain has become a new and attractive target in immunotherapy using monoclonal antibodies [12], IL-13Rα2-peptide pulsed dendritic cells [13], and IL-13R-targeted chimeric antigen receptor modified T cells [14–16]. To target IL-13R, we have developed a recombinant fusion protein composed IL-13 and a mutated form of *Pseudomonas* exotoxin (IL-13-PE) [17]. The IL-13-PE was found to be highly selective and potent in killing human GBM cells in vitro and in animal models of glioma tumors [18–22]. Based on these preclinical studies, several Phase I/II clinical trials targeting IL-13Rα2 in GBM by IL-13-PE were undertaken (https://clinicaltrials.gov). A randomized controlled Phase 3 clinical trial was completed [23] and additional clinical trials are planned.

Despite advances in the understanding of IL-13Rα2 biology in glioma tumors and clinical trials targeting this receptor for therapy, the functional significance of *IL-13Rα2* expression in malignant glioblastoma is not well understood. We and others have shown that IL-13Rα2 may be associated with the increase in glioma malignancy grade and associated with poor patient prognosis [8, 24, 25]. In order to demonstrate a possible correlation between *IL-13Rα1* and *α2* expression in GBM with the clinical outcomes, we analyzed datasets publicly available at NCI’s TCGA database, which was established by NCI/NIH to generate the comprehensive catalog of genomic abnormalities (https://tcga-data.nci.nih.gov/tcga/). The TCGA data provided detailed clinical information of a large number of GBM patients. These datasets were downloaded and an association between expression of *IL-13Rα2* and clinical outcomes in GBM patients was studied. We also examined a possible association between expression of *IL-13Rα1* and clinical outcomes in GBM patients. Our analysis found that the level of *IL-13Rα1* and* α2* expression is associated with poor patient survival, particularly long-term survival and GBM recurrence. Furthermore, some immune regulatory genes seem to be associated with *IL-13Rα2* expression. Our findings have important implications in the understanding of the role of IL-13R in pathogenesis and evaluating possible therapeutic interventions for patients suffering from GBM.

## Materials and methods

### TCGA data description

The publically available TCGA datasets were directly downloaded from the TCGA Data Portal at https://tcga-data.nci.nih.gov/tcga/. The detailed information of the TCGA data structures can be reviewed at https://tcga-data.nci.nih.gov/tcga/tcgaDataType.jsp. The detailed information of the microarray and RNA-Seq experiments, protocols, and software used can be found at the TCGA Data Portal at https://tcga-data.nci.nih.gov/tcga/. For gene expression data, we selected the level-3 microarray dataset, in which Agilent 244K (G4502A), a custom designed microarray platform, was used in the experiments. The microarray data was normalized by Lowess method and presented as calculated Log2 ratio. Additional information regarding the level of the data and methods used in the process can be found at TCGA website (https://tcga-data.nci.nih.gov/tcga/). For RNA-Seq data sets, we selected the level 3 RNA-Seq data which was produced on Illunima HiSeq 2000 sequencers. The RNA-Seq gene expression level 3 data contain Counts which are simply the number of reads overlapping a given gene. The total number of reads for a given transcript is proportional to the expression level of the transcript. Both microarray and RNA-Seq datasets were generated by the laboratories located at University of North Carolina at Chapel Hill. A total of 595 GBM and 10 normal brain microarray data files, 163 RNA-Seq data files, and corresponding clinical data files were downloaded from TCGA website on Sept 27, 2013. These microarray datasets for GBM samples have not changed significantly since they were uploaded at the database.

### Data transformation and classification

The Agilent gene expression microarray data down-loaded from TCGA is presented as log2 ratio of GBM/HuRNA, or Normal brain/HuRNA. In the microarray experiments, Agilent HuRNA (Human universal reference RNA; previously Stratagene HuRNA) was used as a common reference to calculate Log2 ratio. The HuRNA is composed of total RNA pooled from 10 human cancer cell lines. In order to eliminate the potential bias by using the HuRNA as the common reference, we first transformed the original Log2 ratio of GBM/HuRNA to Log2 ratio GBM/Normal brain by using the following formula:

$${\text{Log2 ratio}}\left( {{\text{GBM}}/{\text{normal brain}}} \right)={\text{Log2 ratio}}\left( {{\text{GBM}}/{\text{HuRNA}}} \right)-{\text{Log2 ratio}}\left( {{\text{normal brain}}/{\text{HuRNA}}} \right)$$

$$\begin{aligned} {\text{Since}}:{\text{ }} & {\text{Log2 ratio}}\left( {{\text{GBM}}/{\text{HuRNA}}} \right)-{\text{Log2 ratio}}\left( {{\text{normal brain}}/{\text{HuRNA}}} \right) \\ = & {\text{Log2 ratio}}\left[ {\left( {{\text{GBM}}/{\text{HuRNA}}} \right)/\left( {{\text{normal brain}}/{\text{HuRNA}}} \right)} \right] \\ = & {\text{Log2 ratio }}({\text{GBM}}/{\text{normal brain}}) \\ \end{aligned}$$

Here, the Log2 ratio (normal brain/HuRNA) is the mean value of log2 ratios from the 10 normal brain data files. Then, we used the transformed gene expression data, which was the Log2 ratio of GBM compared to Normal brain, in the rest of our study [26].

In the TCGA datasets, each clinical dataset represented a unique patient case. Survival was defined as the time interval from the date of surgery to the date of death. In order to elucidate a possible correlation between *IL-13Rα2* gene expression and the clinical outcome, we only selected patients with survival > 30 days, indicating that the patient survived from the initial surgery and radiation treatments. A total of 428 GBM gene expression data files having clinical data satisfied the condition for the further gene expression and survival analysis.

**Fig. 1:**
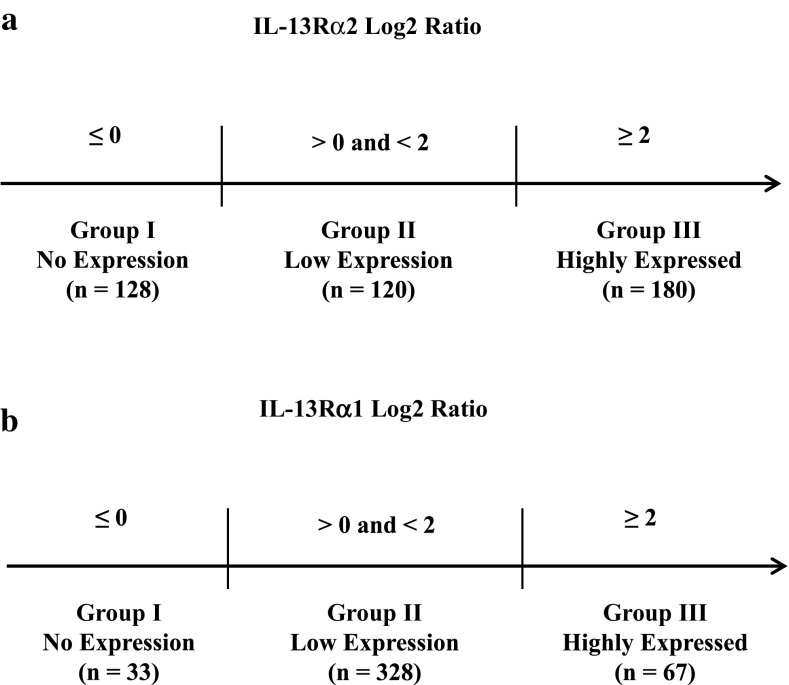
Classification of GBM tumors based on expression analysis of TCGA data base for *IL-13Rα2* and *α1* mRNA: Group I: no expression; Group II: low to moderate expression; and Group III: high expression. **a** *IL-13Rα2* log2 ratio; **b** *IL-13Rα1* log2 ratio

By utilizing the transformed datasets, we classified the TCGA GBM tumors into three groups based on the level of *IL-13Rα2* gene expression. Of 428 GBM tumors studied, 128 cases (29.9%) were classified into group I, which did not express *IL-13Rα2*; 120 cases (28%) were identified in the group II, which expressed *IL-13Rα2* with Log2 ratio of > 0 and < 2; and 180 cases (42.1%) were in the group III, which was defined as the *IL-13Rα2* highly expressed group with Log2 ratio of *IL-13Rα2* ≥ 2 (Fig. 1a).

Same classification was used for *IL-13Rα1* gene expression. Among 428 GBM tumors studied, 33 cases (7.7%) were classified into group I, which did not express *IL-13Rα1*; 328 cases (76.6%) were identified in group II, which expressed *IL-13Rα1* with Log2 ratio of > 0 and < 2; and 67 cases (15.7%) were in group III, which was defined as the *IL-13Rα1* highly expressed group with Log2 ratio of *IL-13Rα1* ≥ 2 (Fig. 1b).

### Statistics analyses

An independent *t* test was performed to calculate the difference between groups. Kaplan–Meier survival analysis was performed to compare the survival distribution between different groups by using GraphPad Prism software (Version 5, GraphPad software Inc., San Diego, CA). A plot of the Kaplan–Meier analysis with appropriate sample size provides the information on the length of survival, median survival time of the distinct sample populations, and significance of the difference between the survival curves.

## Results

### Patient characteristics

We used the publically available GBM dataset in TCGA database as our primary source of clinical information. Each enrolled subject in this dataset had undergone a surgical resection of GBM and received radiation and some form of chemotherapy. Gene expression data was generated from surgical tumor samples. Age distribution of 428 GBM subjects was classified and shown in Supplementary Fig S1. Highest number of patients (56%) is in age ranging between ≥ 50 and < 70-years (Supplementary Fig. S1a). There was a clear gender difference in this age group; the number of male patients with GBM was double compared to number of female patients. However, there was no significant difference between the number of male and female patients in any other age groups (Supplementary Fig. S1b). Patient survival data indicated that 79.2% of patients died within 2 years of diagnosis, including 46.5% of patients died within 1 year. Only about 20% of GBM subjects survived more than 2 years (Supplementary Fig. S2).

### High *IL-13Rα2* expression is associated with poor prognosis

**Fig. 2:**
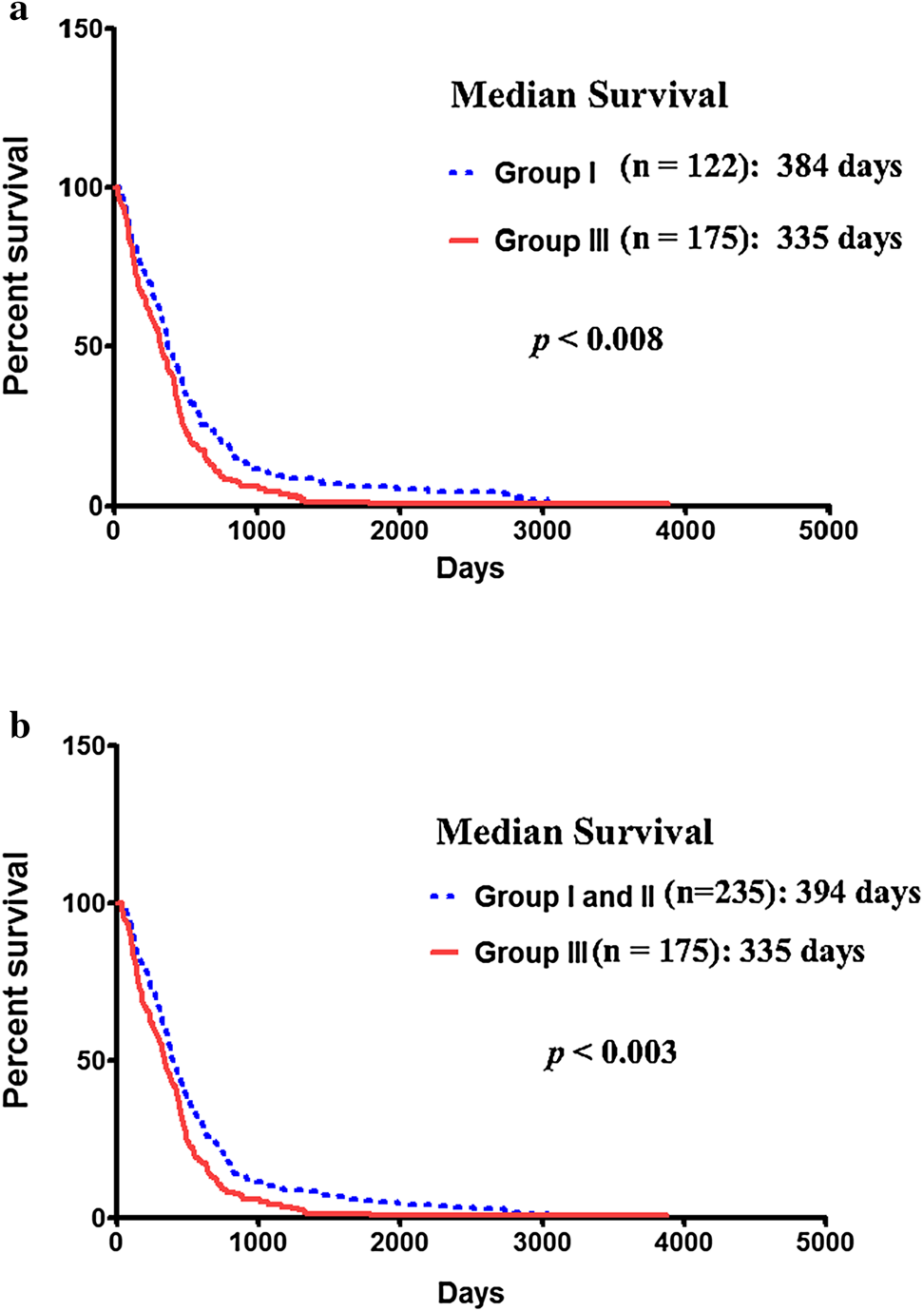
Survival of GBM patients based on *IL-13Rα2* mRNA expression: Kaplan–Meier curve of overall survival for GBM patients segregated based on the gene expression level of IL-13Rα2. Group I represents the *IL-13Rα2* negative, group II represents *IL-13Rα2* low to moderate and group III represents the *IL-13Rα2* high expression. **a** Survival curve comparing Group I and Group III; **b** survival curves comparing Group I and II combined and Group III

To investigate whether *IL-13Rα2* expression is associated with patient prognosis, Kaplan–Meier survival analysis was performed to assess survival of patients with high *IL-13Rα2* and low or no *IL-13Rα2* expressing GBM tumors. A few samples in each group, which have vital status labeled as “Alive”, were eliminated from the dataset for survival analysis. Subjects with GBM that highly expressed *IL-13Rα2* mRNA (Log2 ratio ≥ 2) had a median survival of 335 days (n = 175) compared to 384 days for subjects with *IL-13Rα2* negative tumors (n = 122) (Fig. 2a). The median survival time between *IL-13Rα2* high and *IL-13Rα2* negative groups was statistically significant (*p* < 0.008). However, there was no significant difference in median survival between patients with *IL-13Rα2* low (n = 113) and *IL-13Rα2* negative tumor groups (Supplementary Fig. S3a). Subjects with *IL-13Rα2* low expression had a significantly higher median survival time of 404 days compared to 335 days for subjects with *IL-13Rα2* high tumors (Supplementary Fig. S3b). Furthermore, when subjects with *IL-13Rα2* low (n = 113) and *IL-13Rα2* negative (n = 122) tumor groups were combined together (n = 235), the median survival was significantly higher compared to subjects with high *IL-13Rα2* expressing tumors (*p* < 0.003) (Fig. 2b). These results indicate that subjects with higher level of *IL-13Rα2* expression will have poorer survival demonstrating an inverse relationship between *IL-13Rα2* expression and overall survival.

Although the criteria used to define recurrent GBM remain ambiguous due to the varied presentation of new lesions, we selected the recurrent cases from the “days to tumor recurrence” section listed in the TCGA GBM clinical dataset. We next investigated whether *IL-13Rα2* expression is associated with GBM recurrence. Subjects with highly expressed *IL-13Rα2* (Group III, Log2 ratio ≥ 2, n = 33) had a median days to recurrence of 196 compared to 268 days for subjects with *IL-13Rα2* negative tumors (n = 30) (Supplementary Fig. S4a), although the difference between these two groups was not statistically significant (*p* = 0.065). It is thus possible that subjects with *IL-13Rα2* negative expression will have delayed tumor recurrence compared to subjects with *IL-13Rα2* high expressing tumors and needs to be confirmed in larger sample database.

We also assessed whether *IL-13Rα2* expression was associated with a long-term patient survival. A long-term survival was defined as subject who survived for at least 3 years after diagnosis. A total of 48 subjects survived more than 3 years. Among these, subjects with highly expressed *IL-13Rα2* had a median survival of 1347 days (n = 16) compared to 1796 days for subjects with *IL-13Rα2* negative tumors (n = 14) (Supplementary Fig. S4b). Although the limited numbers of samples prevent achieving statistical significance (*p* = 0.1241), our findings suggest a tendency that patients with *IL-13Rα2* negative tumors may live longer.

When *IL-13Rα2* expression was compared between lower-grade glioma and high-grade GBM, both *IL-13Rα1 and IL-13Rα2* gene expression levels were significantly lower in lower-grade glioma compared with GBM. These results suggest that *IL-13Rα2* gene expression may also be associated with GBM malignancy grade (Supplementary Fig S5).

### High *IL-13Rα2* expression in GBM is associated with potential temozolomide resistance

**Fig. 3:**
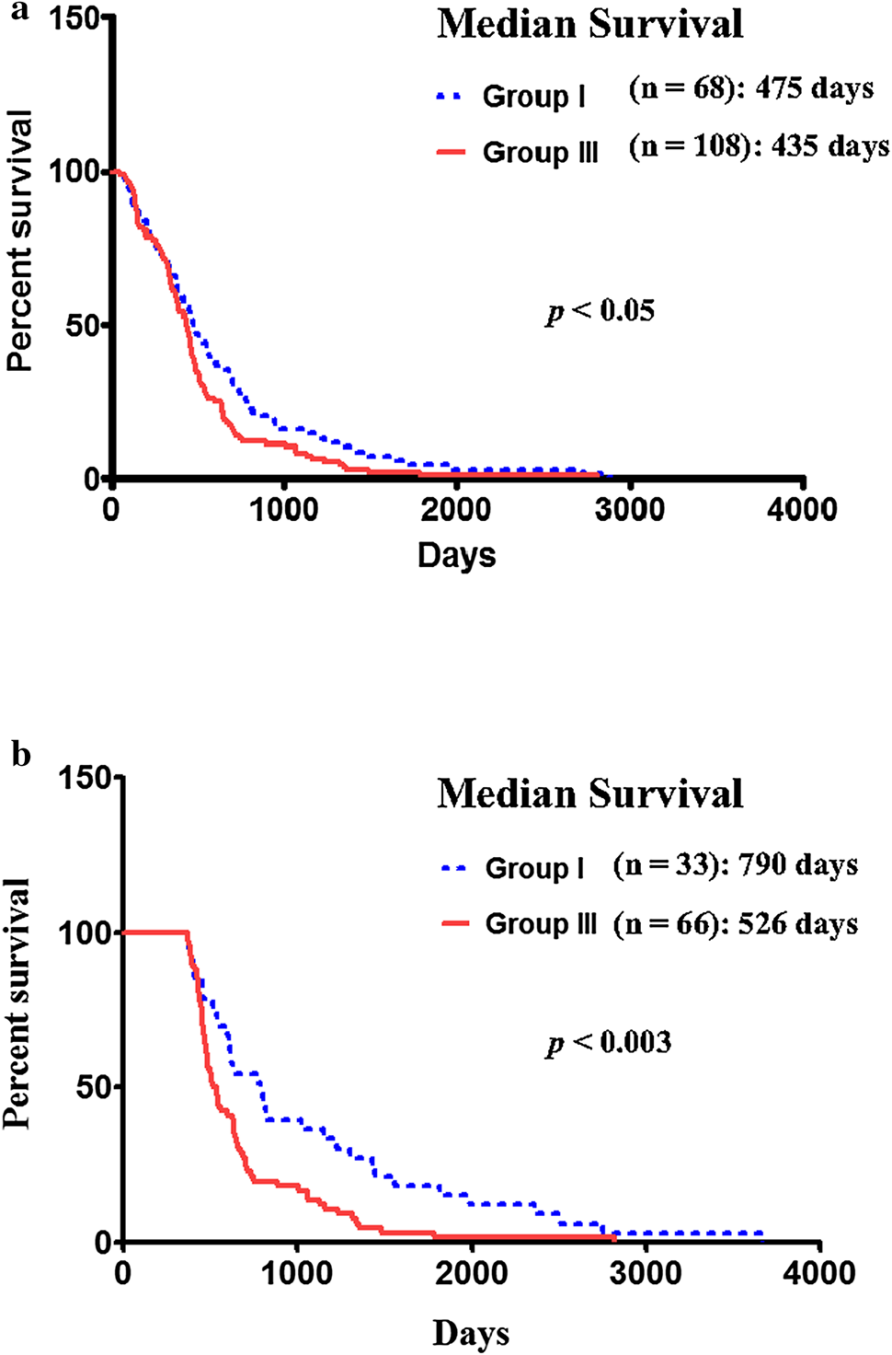
Kaplan–Meier survival curve of GBM patients treated with Temozolomide. **a** Overall survival of patients; and **b** more than 1 year survival based on the gene expression level of *IL-13Rα2*

Temozolomide (TMZ) is a most effective chemotherapeutic agent for GBM. Since *IL-13Rα2* is highly expressed in GBM but not in normal brain, we investigated the potential association between *IL-13Rα2* expression and temozolomide response in GBM patients. We performed Kaplan–Meier analysis to assess the overall survival of subjects with highly expressed *IL-13Rα2* or no expression when treated with temozolomide. Subjects with high *IL-13Rα2* expressing tumors when treated with temozolomide had a median survival of 435 days (n = 108) compared to 475 days (n = 68) in subjects with *IL-13Rα2* negative tumors treated with temozolomide (Fig. 3a). Although the difference between two groups was small, it was statistically significant (*p* < 0.05). However, in subjects with survival time more than 1 year, this difference became clearer. The subjects with highly expressed *IL-13Rα2* (Log2 ratio ≥ 2) had a median survival of 526 days (n = 66) compared to 790 days (n = 33) for subjects with *IL-13Rα2* negative tumors. The difference of the median survival between *IL-13Rα2* high and *IL-13Rα2* negative expression groups was highly statistically significant (*p* < 0.003) (Fig. 3b). In addition, the similar median survival results were obtained between the patients group with *IL-13Rα2* low tumor and the patients group with *IL-13Rα2* high tumor treated with temozolomide (Supplementary Fig. S6a-d). Thus subjects with negative *IL-13Rα2* tumors had better temozolomide responses and significantly longer survival than those subjects with high *IL-13Rα2* expressing tumors. We also performed a detailed data analysis to determine the survival of subjects who did not receive and those who received temozolamide and their GBM tumors expressed high level of *IL-13Rα2* mRNA. However, TCGA database did not have enough number of samples from subjects with high *IL-13Rα2* gene expression who did not receive temozolamide to perform data analysis for statistical significance. Overall, our findings suggest that *IL-13Rα2* over expression is associated with temozolomide resistance in GBM patients.

### Expression of *IL-13Rα1* and *IL-4Rα* and their correlations with *IL-13Rα2* in GBM

**Fig. 4:**
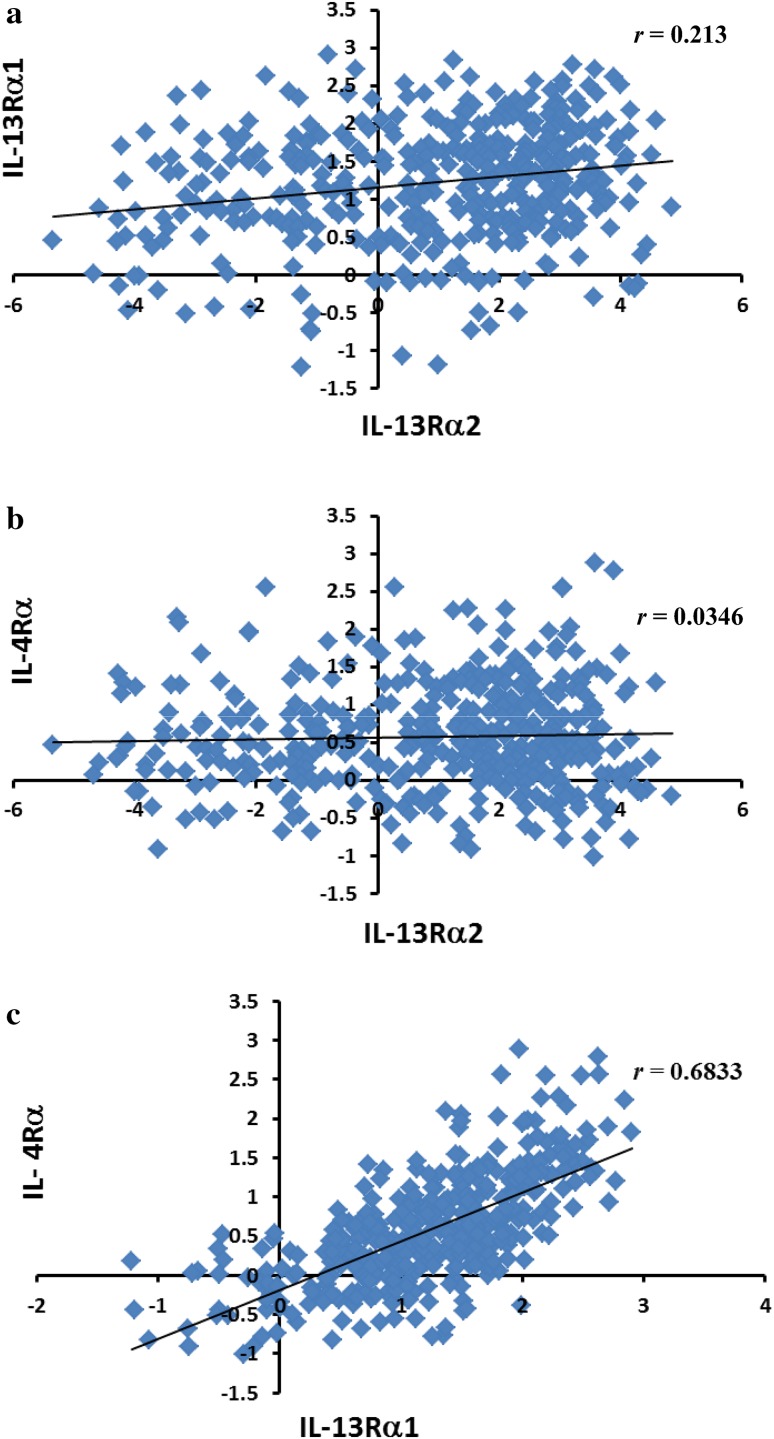
Correlation between IL-13R subunits mRNA. Gene expression of *IL-13Rα2* and *IL-13Rα1, IL-13Rα2* and *IL-4Rα*, and *IL-13Rα1* and *IL-4Rα* was correlated. **a** Correlation between gene expression of *IL-13Rα2* and *IL-13Rα1*; **b** correlation between gene expression of *IL-13Rα2* and *IL-4Rα*; and **c** correlation between gene expression of *IL-13Rα1* and *IL-4Rα*. Where *r* is a correlation coefficient

Three different types of IL-13 receptors have been reported [27, 28]. In type I IL-13R, three chains (IL-13Rα1, IL-13Rα2, and IL-4Rα) are present that bind IL-13. Type II IL-13R consists of IL-13Rα1 and IL-4Rα chains. In type III IL-13R, an additional component of IL-2Rγ (common γ chain, γc) is present. We first investigated whether the expression of IL-13 receptor chains (*IL-13Rα1, IL-13Rα2*, and *IL-4Rα*) in GBM correlated with each other. Although mRNA for three chains of IL-13 receptor are expressed at different levels in GBM samples, we did not find any correlation between the expression of *IL-13Rα2* and of *IL-13Rα1* (Fig. 4a) mRNA, nor any correlation between the expression of *IL-13Rα2* and of *IL-4Rα* mRNA (Fig. 4b). In contrast, the expression of *IL-13Rα1* mRNA positively correlated with *IL-4Rα* indicating that these two receptors may form a complex in GBM (Fig. 4c). Furthermore, although low level *IL-2Rγ* mRNA was also detected, no correlation between the expression of *IL-2Rγ* (common γ chain, γc) and either the expression of *IL-13Rα1*, or of *IL-13Rα2* was detected, except week correlation with *IL-4Rα* expression in GBM (Supplementary Fig. S7a-c).

We further investigated the possible correlation between expressions of cytokine IL-13, IL-4 and IL-13 receptors. We found that there was no correlation between expression of IL-13 and of IL-13 receptors (Supplementary Fig. S7d, f) and no correlation between expression of IL-4 and IL-13 receptors (Supplementary Fig. S7e, g). Since IL-4 and IL-13 are predominantly produced by Th2 and other cells but not GBM [29], this observation was not unexpected. Furthermore, the data indicated that there was no possible IL-13 or IL-4 autocrine signaling involved in GBM tumors.

### High *IL-13Rα1* expression is also associated with poor patient prognosis

**Fig. 5:**
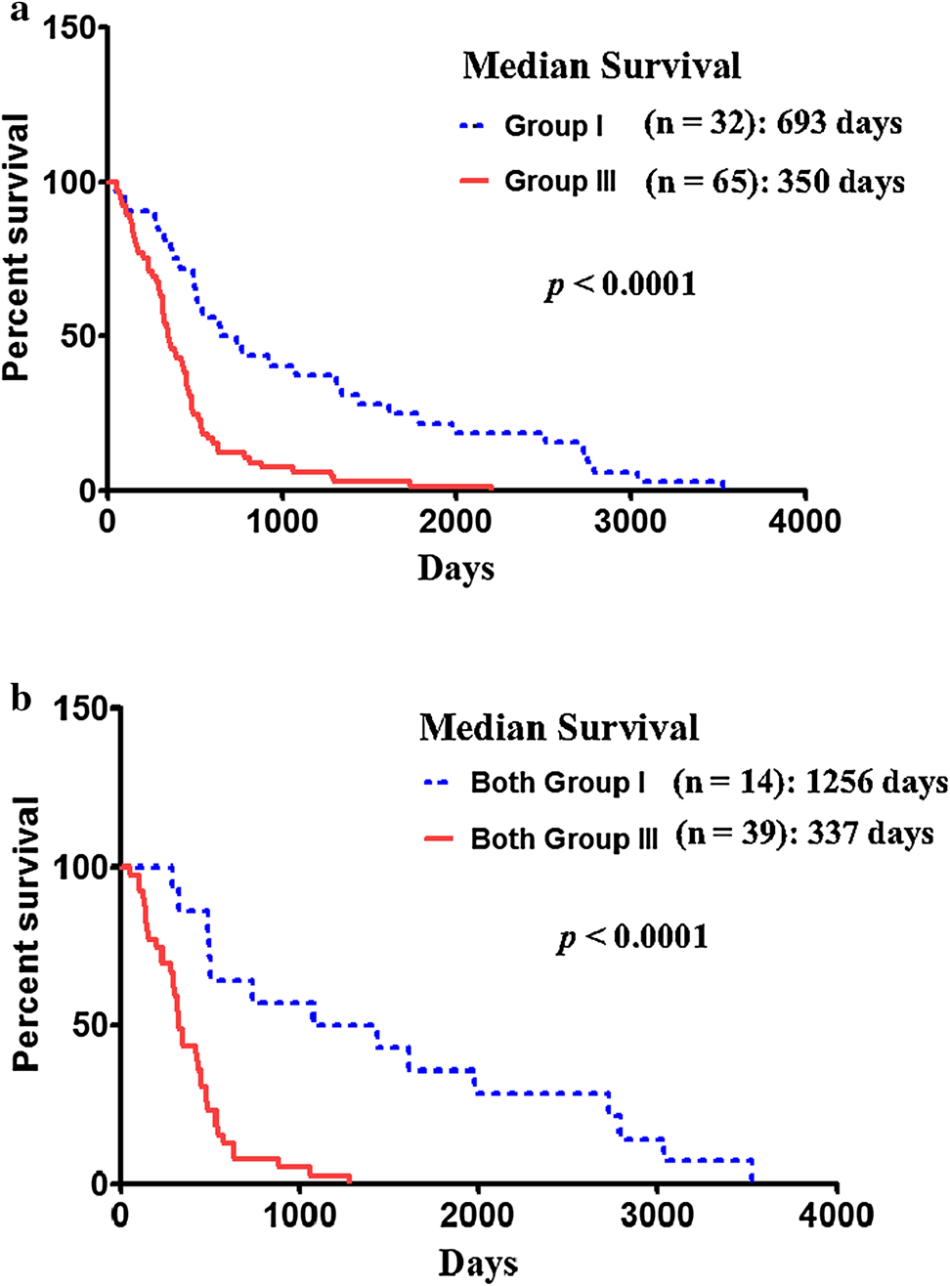
Kaplan Meier survival curve of GBM patients based on the expression of *IL-13Rα1* mRNA. **a** Overall patient survival based on the expression level of *IL-13Rα1* mRNA; Group I: *IL-13Rα1* negative; Group III: *IL-13Rα1* highly expressed (2 < Log2); **b** overall patient survival based on both *IL-13Rα1* and *IL-13Rα2* expression; both Group I: *IL-13Rα1* and *IL-13Rα*2 negative; both Group III: *IL-13Rα1* and *IL-13Rα2* highly expressed (2 < Log2)

To investigate whether *IL-13Rα1* mRNA expression is also associated with patient prognosis, Kaplan–Meier survival analysis was performed to assess survival of subjects with high *IL-13Rα1* (Log2 ratio > 2) and no *IL-13Rα1* mRNA (Log2 ratio < 0) expressing GBM tumors. Subjects with highly expressed *IL-13Rα1* mRNA had a median survival of 350 days (n = 65) compared to 693 days for subjects with no *IL-13Rα1* mRNA tumors (n = 32) (Fig. 5a). The median survival time between *IL-13Rα1* high and *IL-13Rα1* negative groups was highly statistically significant (*p* < 0.0001).

Although we did not find any correlation between *IL-13Rα1* mRNA and *IL-13Rα2* mRNA expression, we investigated whether both *IL-13Rα1* and *IL-13Rα2* expression combined is associated with patient prognosis. Subjects with both *IL-13Rα1* and *α2* high expression (both Log2 ratio > 2) had a median survival of 337 days (n = 39) compared to 1256 days for subjects with both *IL-13Rα1* and *α2* negative tumors (n = 14) (Fig. 5b). The median survival time between both *IL-13Rα1* and *α2* high and both *IL-13Rα1* and *α2* negative group was highly statistically significant (*p* < 0.0001). Subjects with both *IL-13Rα1* and *α2* high expressions will have poorest survival rate in GBM patient population.

### Verification of gene expression data from microarray technology by RNA-Seq technology

**Fig. 6:**
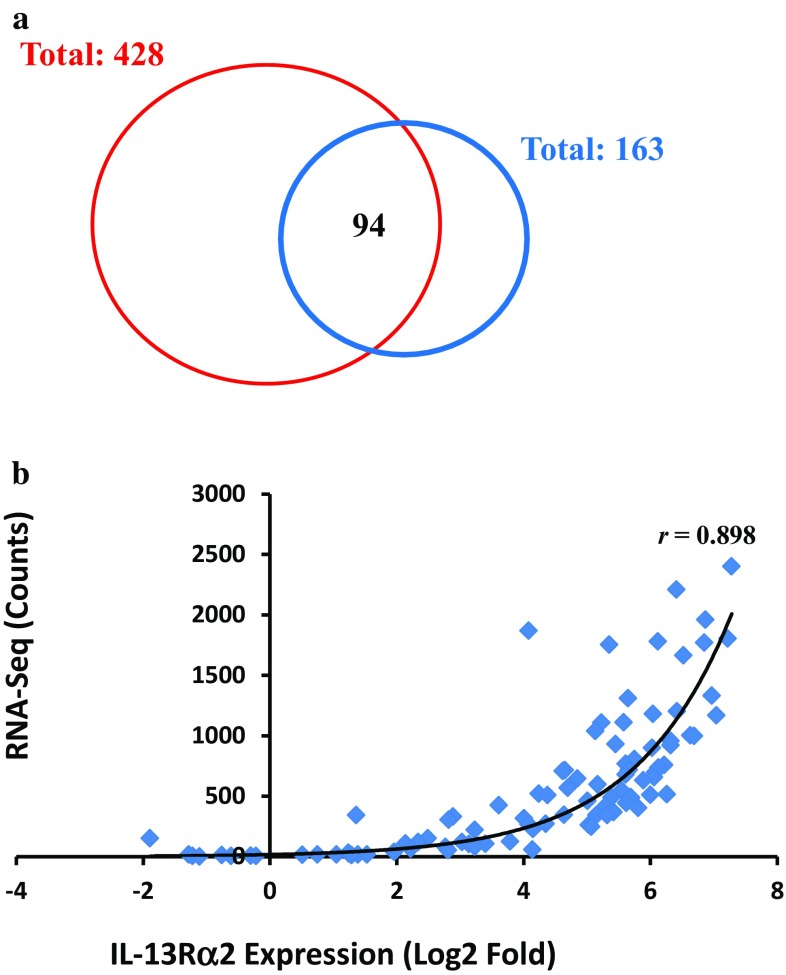
Venn diagram of dataset distribution of gene expression based on RNA-Seq data analysis. **a** A total of 428 gene expression files and 163 RNA-Seq data files were analyzed from the TCGA dataset. There were only 94 GBM tumors with both gene expression and RNA-Seq datasets. **b** The expression of *IL-13Rα2* gene from microarrays showed a strong positive correlation with the counts of *IL-13Rα2* gene detected by RNA-Seq technology (*r* = 0.898), indicating that the transformed Log2 ratio data for gene expression was highly reliable

RNA-Seq provides a far more precise measurement of levels of transcripts than any other methods [30]. A total of 163 RNA-Seq data files were downloaded from TCGA, and only 94 data files overlapped with microarray gene expression files, meaning that only 94 GBM tumors in TCGA have both microarray and RNA-Seq datasets (Fig. 6a). Even though there was only a limited amount of RNA-Seq data available in GBM dataset, we used the RNA-Seq data to evaluate the reliability of our transformed microarray gene expression data. We found that the expression of *IL-13Rα2* gene from microarrays showed a strong positive correlation with the counts of reads that align with the *IL-13Rα2* gene detected by RNA-Seq technology. *IL-13Rα2* expression was strongly correlated (*r* = 0.898) between the microarray and RNA-Seq data indicating that the transformed Log2 ratio was very reliable, and represented the alterations of genes in GBM compared to the normal brain tissue (Fig. 6b).

### Analyses of immune regulatory genes associated with *IL-13Rα2* expression

In order to investigate the possible connection of immune genes with *IL-13Rα2* expression in GBM, we selected the Immport Gene List, which contained 4815 immune-associated genes, from InnateDB (http://www.innatedb.com), which is an integrated analysis platform that has been specifically designed to facilitate systems-level analyses of mammalian innate immunity networks, pathways and genes [31]. We analyzed expression of immune associated genes in *IL-13Rα2* highly expressed GBM group and compared with *IL-13Rα2* negative GBM group. A total of 132 genes were identified that were overexpressed in *IL-13Rα2* highly expressing tumors (Table 1 and Supplementary Table S1).

**Table 1:** Selected immune related genes expressed only in IL-13Rα2 mRNA highly expressed GBM tumors (Group III)

Gene symbol	Gene name	Log2 ratio^a^
FMOD	Fibromodulin	1.92
CXCL13	Chemokine (C–X–C motif) ligand 13	1.61
MNDA	Myeloid cell nuclear differentiation antigen	1.58
HLA-DRA	Major histocompatibility complex, class II, DR alpha	1.52
TLR8	Toll-like receptor 8	1.50
LY75	Lymphocyte antigen 75	1.48
IL15	Interleukin 15	1.48
IL18	Interleukin 18 (interferon-gamma-inducing factor)	1.45
CCR5	Chemokine (C–C motif) receptor 5 (gene/pseudogene)	1.43
CCL2	Chemokine (C–C motif) ligand 2	1.37
ITGBL1	Integrin, beta-like 1 (with EGF-like repeat domains)	1.36
OSM	Oncostatin M	1.36
WISP1	WNT1 inducible signaling pathway protein 1	1.35
TREM2	Triggering receptor expressed on myeloid cells 2	1.35
SRGN	Serglycin	1.33
GJB6	Gap junction protein, beta 6, 30 kDa	1.33
MMRN1	Multimerin 1	1.25
FYB	FYN binding protein	1.25
PLA2G4A	Phospholipase A2, group IVA	1.23
IL17B	Interleukin 17B	1.23
TLR6	Toll-like receptor 6	1.23
TLR5	Toll-like receptor 5	1.23
AIF1	Allograft inflammatory factor 1	1.21
FOXJ1	Forkhead box J1	1.20
TSPO	Translocator protein (18 kDa)	1.20
GZMK	Granzyme K (granzyme 3; tryptase II)	1.20
THBD	Thrombomodulin	1.19
SULF1	Sulfatase 1	1.18
IFI6	Interferon, alpha-inducible protein 6	1.17
BTG3	B cell translocation gene 3	1.16
SERPING1	Serpin peptidase inhibitor, clade G (C1 inhibitor), member 1	1.15
SYK	Spleen tyrosine kinase	1.14
CSF3R	Colony stimulating factor 3 receptor (granulocyte)	1.13
TGFBR1	Transforming growth factor, beta receptor 1	1.12
PSMB10	Proteasome (prosome, macropain) subunit, beta type, 10	1.12
SIPA1	Signal-induced proliferation-associated 1	1.11
CIITA	Class II, major histocompatibility complex, transactivator	1.11
LILRB1	Leukocyte immunoglobulin-like receptor, subfamily B 1	1.11
TNFRSF14	Tumor necrosis factor receptor superfamily, member 14	1.11
TIPARP	TCDD-inducible poly(ADP-ribose) polymerase	1.10
LCP2	Lymphocyte cytosolic protein 2	1.10
FPR1	Formyl peptide receptor 1	1.10
CFD	Complement factor D (adipsin)	1.10
ATF5	Activating transcription factor 5	1.10
ANXA4	Annexin A14	1.09
LCP1	Lymphocyte cytosolic protein 1 (l-plastin)	1.09
CFH	Complement factor H	1.09
OXTR	Oxytocin receptor	1.08
IL33	Interleukin 33	1.08
CD226	CD226 molecule	1.08
F3	Coagulation factor III (thromboplastin, tissue factor)	1.07
CFLAR	CASP8 and FADD-like apoptosis regulator	1.07
IL7R	Interleukin 7 receptor	1.03
BCAP29	B cell receptor-associated protein 29	1.03
IFI35	Interferon-induced protein 35	1.02
MAGED1	Melanoma antigen family D, 1	1.02
IFT52	Intraflagellar transport 52 homolog (chlamydomonas)	1.02
CD276	CD276 molecule	1.02
TBXAS1	Thromboxane A synthase 1 (platelet)	1.02
CXCR4	Chemokine (C–X–C motif) receptor 4	1.01

^a^Log2 ratios are the mean values of immune related genes expressed in IL-13Rα2 highly expressed tumor group (Group III)

Among immune associated genes, we looked at the expression of immunosuppressive genes. We found that *FMOD* (fibromodulin), *CCR5* (chemokine receptor), and *OSM* (oncostatin M) genes were highly expressed in *IL-13Rα2* highly expressed group, but not in *IL-13Rα2* negative group. *FMOD* is associated as a risk factor of poor GBM prognosis [32], while *CCR5*, as a cell surface receptor, plays a role in cancer cell proliferation, metastasis, and the formation of an immunosuppressive microenvironment [33]. Oncostatin M (*OSM*), a cytokine belonging to the interleukin-6 family, has been shown to be increased in a variety of cancers, including malignant glioma. *OSM* induces *ADM* (Adrenomedullin) expression in astroglioma cells through induction of signal transducer and activator of transcription-3 (STAT-3) phosphorylation, which is involved in glioma development and progression [34].

*CCL2* (chemokine (C-C motif) ligand 2) and *CXCL13* (chemokine (C-X-C motif) ligand 13) genes are also highly expressed in the *IL-13Rα2* high group. The role of *CCL2* and its receptors in the attraction of monocytic myeloid-derived suppressor cells (MDSCs) has been reported. An accumulation of monocytic MDSCs in tumors occurred via an interaction between *CCL2* and its receptors. Monocytic MDSCs exhibited *CCR2*-dependent immunosuppressive activities. The production of *CCL2* led to the MDSC accumulation in cancer environment [35].

## Discussion

We demonstrate that GBM tumors can be classified into three different distinct groups based on the analysis of gene expression data from 428 glioma subjects at the TCGA database for *IL-13R* (*α1* and *α2*) gene expression. Group I tumors do not express *IL-13Rα1* and *α2* mRNA; group II tumors expressed mild to moderate levels of *IL-13Rα1* and *α2* mRNA, while groups III tumors expressed high level of *IL-13Rα1* and *α2* mRNA. A large % of GBM samples (76%) expressed mild to moderate levels (Log2 > 0 to < 2) of *IL-13Rα1*, while 28% samples expressed mild to moderate levels of *IL-13Rα2* mRNA. More than 42% of GBM samples were highly positive for *IL-13Rα2* mRNA (Log2 ≥ 2) while only 16% samples were highly positive for *IL-13Rα1* mRNA. Patients with group III tumors had the shortest overall survival irrespective of treatment compared to group I and group II patients. Thus overexpression of *IL-13Rα1* and *α2* gene expression in tumors is associated with decreased patient survival and poor patient prognosis. In addition, patients with highest expression of both *IL-13Rα1* and *α2* mRNA in tumors showed poorest survival in GBM patients. *IL-13Rα2* mRNA expression was confirmed by RNA-seq technology. These results indicate that *IL-13R* expression in glioma tumors is associated with poor patient prognosis and it is possible that IL-13Rs are prognostic indicator for GBM. Furthermore, when *IL-13Rα2* expression was compared between lower-grade glioma and high grade GBM, both *IL-13Rα1 and IL-13Rα2* gene expression levels were significantly lower in lower-grade glioma compared with GBM. These results suggest that *IL-13Rα2* gene expression may also be associated with GBM malignancy grade.

Temozolomide-based therapy is the standard of care for patients with GBM. However, the efficacy of standard temozolomide chemotherapy and radiation therapy for patients with GBM is compromised by resistance to these therapies. In vitro studies demonstrated that *CD133* positive GBM cells show strong tumor’s resistance to chemotherapeutic agents, including TMZ [36]. It was found that expression levels of *CD74* in high grade gliomas were inversely associated with TMZ resistance in GBM xenograft lines, suggesting a role in TMZ resistance [37]. In addition, the resistance to TMZ has been shown to be modulated by the DNA repair protein *O*6-methylguanine-DNA methyltransferase (*MGMT*). It has been shown that elevated *MGMT* protein levels or lack of *MGMT* promoter methylation is associated with TMZ resistance in some, but not all GBM tumors [38]. Although DNA methylation of the MGMT gene promotor was shown to be a prognostic marker for treatment response of temozolomide in GBM [39], Brennan et al. reported that *MGMT* status distinguishes responders from non-responders to TMZ only among samples classified as classical subtype of GBM (n = 96), but not among other samples classified as proneural, mesenchymal, and neural subtypes of GBM (a total of n = 225) [40]. Their data indicate that *MGMT* DNA methylation can only be used as a prognostic marker for the classical subtype of GBM, but not for any other subtypes of GBM [40]. In our study, we observed for the first time that the patients with over expressed *IL-13Rα2* treated with TMZ chemotherapies had shorter overall survival time compared with the patients with *IL-13Rα2* negative expression treated with TMZ, implicating that *IL-13Rα2* mRNA expression is associated with GBM resistance to TMZ chemotherapy. In addition, we did not find any correlation of *IL-13Rα2* mRNA expression with the *MGMT* expression (Supplementary Table S2), indicating that the influence of *IL-13Rα2* expression on TMZ response was independent of the expression of *MGMT*. These data suggest that *IL-13Rα2* may be a new modulator of TMZ response, representing a distinct mechanism of TMZ resistance from *MGMT*.

Targeting *IL-13Rα2* has motivated the development of highly effective therapies and novel administration strategies. So far, a total of six clinical trials using IL-13-PE in patients with various malignant gliomas have been completed in the United States (https://clinicaltrials.gov). Early clinical trials (Phase I) targeting *IL-13Rα2*, by IL-13-PE38QQR via CED in combination with the current standard of care (surgery, radiotherapy, and temozolomide) showed promising safety and efficacy profiles (http://clinicaltrials.gov). A completed phase III randomized clinical trial of convection enhanced delivery (CED) of IL-13-PE38QQR vs. an FDA approved drug carmustine-releasing Gliadel wafers (GW) for recurrent glioblastoma, showed that IL-13-PE was well tolerated, but it did not show superiority over GW in overall survival. Retroactive data analysis of time-to-progression was significantly higher with IL-13-PE compared to GW [23]. However, tumor specimens from the original surgery were not evaluated for the presence of IL-13 receptors in the enrolled patients in this trial. The outcome could potentially be improved if enrolled patients could be limited only to ones with over expressed IL-13Rα2 in tumor tissue. Furthermore, IL-13-PE treatment data in the TCGA dataset showed that IL-13-PE treated patients had a longer median survival (657 days) compared with median survival of the reference patient group without IL-13-PE treatment (384 days) (Supplementary Fig S8). Although the sample size of IL-13-PE treated patients in TCGA was extremely small (n = 6), these preliminary results have significant implications and indicate that targeting of *IL-13Rα2* in GBM treatment holds a promise, specifically for the *IL-13Rα2* positive group of patients.

The mechanism of poor survival of patients with GBM tumors expressing high levels of *IL-13Rα1* and *IL-13Rα2* is not clear. *IL-13* and *IL-13Rα2* has been shown to be involved in immune evasion and tolerance mechanisms and thus it is possible that high *IL-13Rα1* and *IL-13Rα2* expression participates in systemic profound immunosuppression seen in GBM patients [41]. In that regard, we found that several immunosuppressive genes were highly expressed in *IL-13Rα2* over expressed tumors, but not in *IL-13Rα2* negative tumors. These genes included *CCL2*, which is known to attract MDSCs in cancer microenvironment. MDSCs represent one of the most important players mediating immunosuppression. These cells may not only inhibit an anti-tumor immunity but also directly stimulate tumorigenesis as well as tumor growth and expansion [35]. MDSCs reduce antigen specific CD8 + T cell proliferation, increase T-cell death by apoptosis, and change the profile of cytokines secreted by activated T lymphocytes [42]. It is possible that targeting MDSCs will have a favorable outcome in patients with GBM. A better understanding of the contribution of the tumor on systemic immune suppression is necessary for improved therapies, to monitor negative effects of novel treatments, and to improve patient outcomes.

Recently, various gene expression studies have identified gene signatures that are associated with various types of GBM as well as signatures that correlate with survival. Kim et al. identified 42 probe sets that show an association with tumor aggressiveness and patient survival [5]. Verhaak et al. identified a gene signature associated with four subtypes (proneural, neural, classical, and mesenchymal) of GBM. An 840 gene signature (210 genes per class) was established. Each of the signatures was highly distinctive [4]. These defined subtypes differ by the type of genetic abnormalities they carried and by the patient’s clinical characteristics. A high level of *EGFR* expression and *EGHR* amplification were mainly observed in the classical subtypes. The *IDH1* and *TP53* mutations were significantly frequent events in the proneural subtype. *IDH1* somatic mutation has been linked to a glioma-CpG island methylator phenotype (G-CIMP) [43]. Turcan et al., have demonstrated that *IDH1* mutation is the cause of CIMP and leads to CIMP phenotype, and is sufficient to establish the glioma hypermethylator phenotype [44]. G-CIMP tumors belong to the proneural subgroup in GBM and are more prevalent among lower-grade gliomas [43, 45]. In addition, *PDGFRA* was another gene, which was mutated and highly expressed only in the proneural subtype [4]. Brown et al. reported that high *IL-13Rα2* gene expression is positively correlated with the mesenchymal signature gene expression and negatively correlated with the proneural signature gene expression [24]. They further showed that *IL-13Rα2* expression is correlated, but not limited to the expression of mesenchymal signature genes. In our study, we did not find any correlation between *IL-13Rα2* mRNA expression and previously reported biomarkers of GBM subtypes such as IDH1, EGFR, MGMT, and PDGFRA (Supplementary Table S2). Furthermore, we also found that high expression of *IL-13Rα1* and *α2* genes are associated with poor prognosis in *IDH1*-Wt/non-G-CIMP GBM (Supplementary Fig S9a and b). Our Data shows that approximately 70% GBM tumors express moderate to high level of *IL-13Rα2* mRNA, which is expressed in most of the subtypes identified by Verhaak et al. [4]. Our recent studies in animals confirm our observations that *IL-13Rα2* is involved in tumor invasion, metastasis and poor survival of animals implanted with human pancreatic and ovarian cancers [9, 10]. These results indicate that a single gene (*IL-13Rα2*) may provide a stronger correlation with survival than a group of genes previously identified, thus making *IL-13Rα2* an important target for glioma therapy.

It is of interest to note that mRNA for three chains of IL-13 receptor (*IL-13Rα2, IL-13Rα1* and *IL-4Rα*) are expressed at different levels in GBM samples. *IL-13Rα2* was highly expressed in more than 42% of total GBM samples, compare with that of *IL-13Rα1* only highly expressed in less than 16% of the total GBM. We did not find any correlation between the expression of *IL-13Rα2* and *IL-13Rα1*, nor between *IL-13Rα2* and *IL-4Rα* mRNA. These analyses indicate that over expression of *IL-13Rα2* mRNA in GBM is independent from expression of *IL-13Rα1* or *IL-4Rα* suggesting that IL-13Rα2 does not seem to form a complex with either IL-13Rα1 or IL-4Rα chain. This is consistent with our previous work that summarized by Suzuki et al. and Joshi et al. [29, 46]. In contrast, the expression of IL-13Rα1 mRNA positively correlated with IL-4Rα indicates that these two receptor chains form a type II IL-13R complex in GBM. Indeed, this receptor has been shown to mediate IL-13 signaling in cancer cells [7, 10, 29].

In conclusion, we have found that high *IL-13Rα1* and *IL-13Rα2* mRNA expression is associated with poor patient prognosis, demonstrating an inverse relationship between IL-13R expression and overall survival. The similar inverse relationship seems to be also associated with days to GBM recurrence and long-term patient survival. Furthermore, we show for the first time that IL-13Rα2 expression is associated with GBM resistance to TMZ chemotherapy. These findings have important implications in understanding a possible role of IL-13R in GBM pathogenesis, development of targeted therapies, and define a patient population for immunotherapy or alternative therapies in clinical trials. Additional studies are ongoing to further confirm our observations.

## Electronic supplementary material

Below is the link to the electronic supplementary material.

Supplementary material 1 (DOC 39 KB)

Supplementary material 2 (PDF 183 KB)

Supplementary material 3 (DOC 34 KB)

Supplementary material 4 (PDF 71 KB)
