# Supplementary material for: Analysis of the cancer genome atlas (TCGA) database identifies an inverse relationship between interleukin-13 receptor α1 and α2 gene expression and poor prognosis and drug resistance in subjects with glioblastoma multiforme
Source: J Neurooncol. 2017 Nov 22;136(3):463–74. doi: 10.1007/s11060-017-2680-9 (PMC5805806; doi:10.1007/s11060-017-2680-9)
Supplement: Supplementary file 1 — Supplementary material 1 (DOC 39 KB) [file 11060_2017_2680_MOESM1_ESM.doc]

**Supplementary Figure Legend**

**Fig. S1** Characteristics of TCGA GBM tumors. **a** Numbers of GBM patients in different age groups; **b** Gender differences of GMB patients in different age groups.

**Fig. S2** Percentages of survivals in GBM patients in each time group (year).

**Fig. S3** Survival of GBM patients based on *IL-13Rα2* mRNA expression: Kaplan-Meier curve of overall survival for GBM patients segregated based on the gene expression level of *IL-13Rα2*. Group I represents the *IL-13Rα2* negative, group II represents *IL-13Rα2* low expression, and group III represents the *IL-13Rα2* high expression. **a** Survival curves between Group I and Group II; **b** Survival curves between Group II and Group III.

**Fig. S4** Kaplan-Meier curve of overall survival. **a** Stratification of GBM recurrence. Group I represents the *IL-13Rα2* negative group, and group III represents the *IL-13Rα2* high group. **b** Long-term survivors segregated based on the gene expression level of *IL-13Rα2*.

**Fig. S5** Comparison of *IL-13Rα1* and *α2* expression in lower-grade glioma and high grade GBM. **a** Comparison of *IL-13Rα2* expression between lower-grade glioma and high grade GBM; **b** *IL-13Rα1* and *α2* expression in lower-grade glioma. LGG = low grade glioma

**Fig. S6** Kaplan-Meier survival curves of GBM patients treated with Temozolomide. **a** Survival curves between Group I and Group II; **b** Survival curves between Group II and Group III; **c** Overall survival between Group I/II and Group III patients; and **d** more than one year survival between Group I/II and Group III patients.

**Fig. S7** Correlations between IL-13R, IL-4R subunits, and IL-13 and IL-4 mRNAs. **a** Correlation between expression of *IL-2Rγ* and *IL-13Rα1*; **b** Correlation between expression of *IL-2Rγ* and *IL-13Rα2*. **c** Correlation between expression of *IL-2Rγ* and *IL-4Rα*; **d** Correlation between expression of *IL-13Rα1* and *IL-13*; **e** Correlation between expression of *IL-13Rα1* and *IL-4*; **f** Correlation between expression of *IL-13Rα2* and *IL-13*; **g** Correlation between expression of *IL-13Rα2* and *IL-4*; **h** Correlation between expression of *IL-4Rα* and *IL-13*; **i** Correlation between expression of *IL-4Rα* and *IL-4*.

**Fig. S8** Kaplan-Meier curves of survival for GBM patients treated with IL-13-PE, compared with the patients in Group I as a reference.

**Fig. S9** Survival of subjects with GBM tumors expressing *IL-13R* mRNA in *IDH1*-Wt group: Kaplan-Meier curve of overall survival segregated based on the gene expression level of *IL-13Rα2 and α1, after removing IDH1 mutated samples from the dataset.* **a.** Group I represents the *IL-13Rα2* negative, and group III represents the *IL-13Rα2* high expression (Log2≥2), survival curves comparing Group I and Group III; **b.** Survival based on both *IL-13Rα1* and *IL-13Rα2* expression; Both Group I: *IL-13Rα1* and *IL-13Rα*2 negative; Both Group III: *IL-13Rα1* and *IL-13Rα2* highly expressed (Log2≥2).
