# Supplementary figures and images for: Analysis of the cancer genome atlas (TCGA) database identifies an inverse relationship between interleukin-13 receptor α1 and α2 gene expression and poor prognosis and drug resistance in subjects with glioblastoma multiforme
Source: J Neurooncol. 2017 Nov 22;136(3):463–74. doi: 10.1007/s11060-017-2680-9 (PMC5805806; doi:10.1007/s11060-017-2680-9)

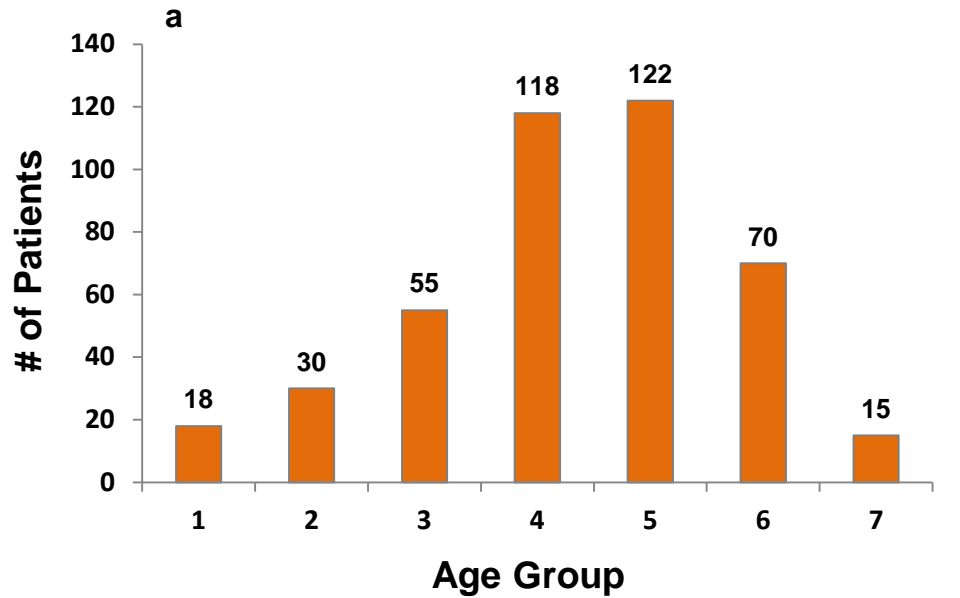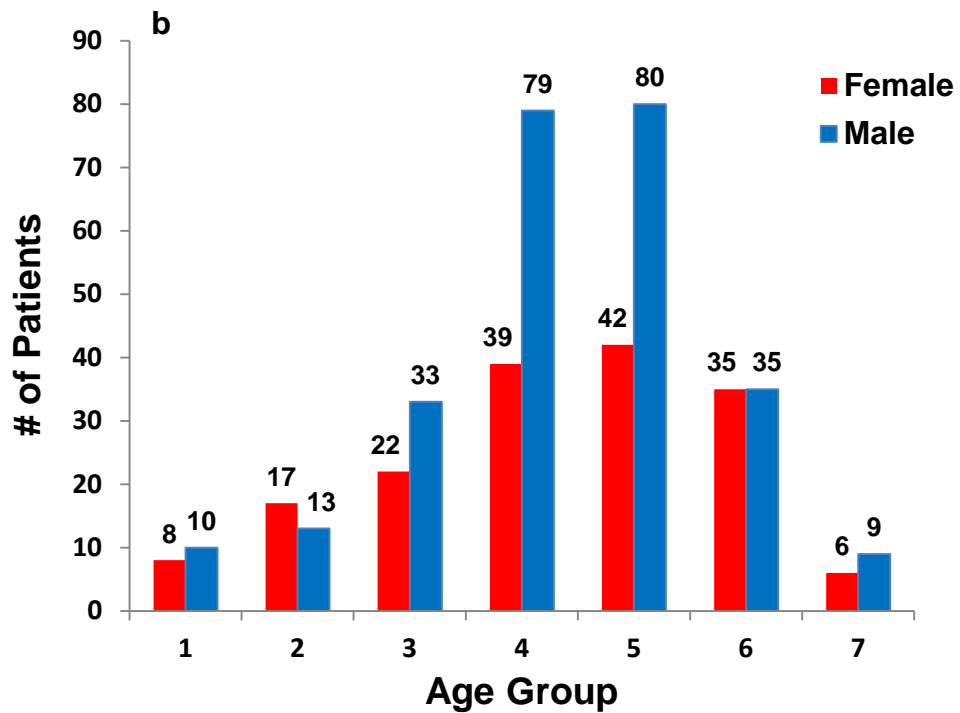

**Age Group:**

1. < 30 years

4. 50 ≤ and < 60

7. > 80

2. 30 ≤ and < 40

5. 60 ≤ and < 70

3. 40 ≤ and < 50

6. 70 ≤ and < 80

**Fig S1**

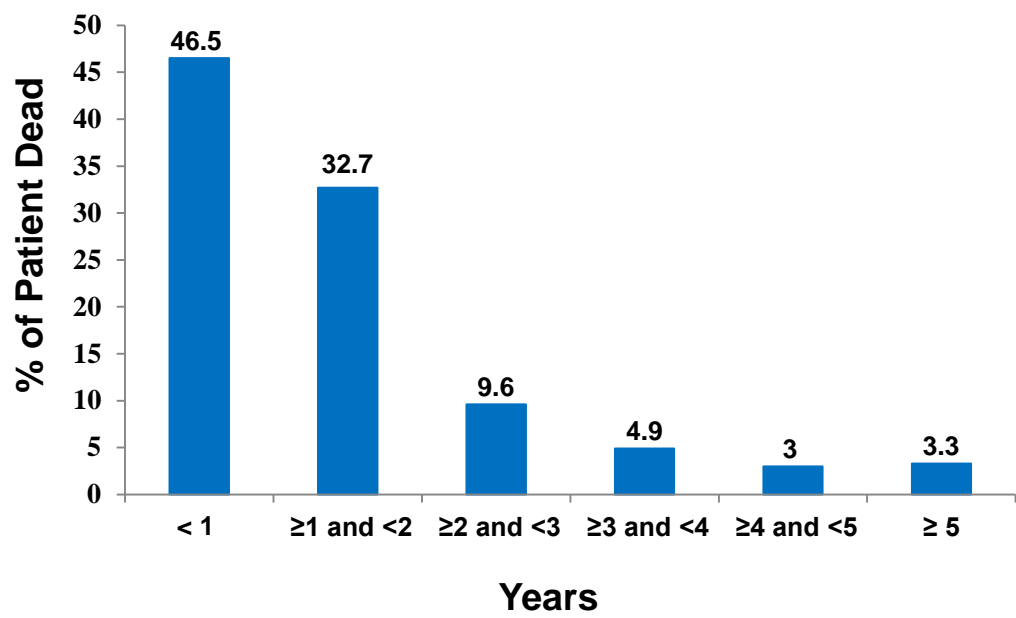

**Fig S2**

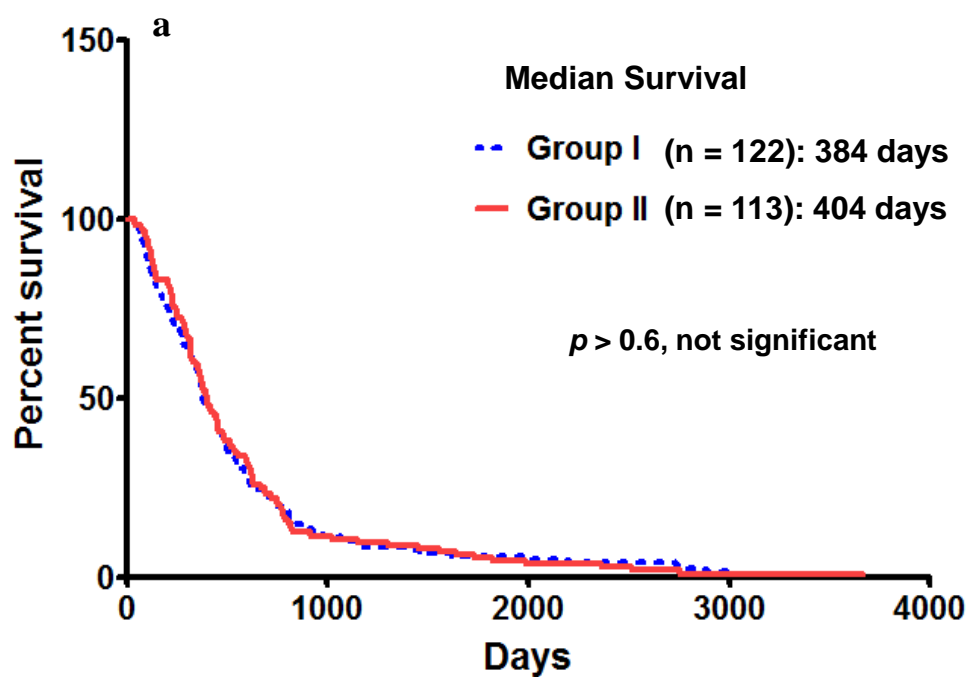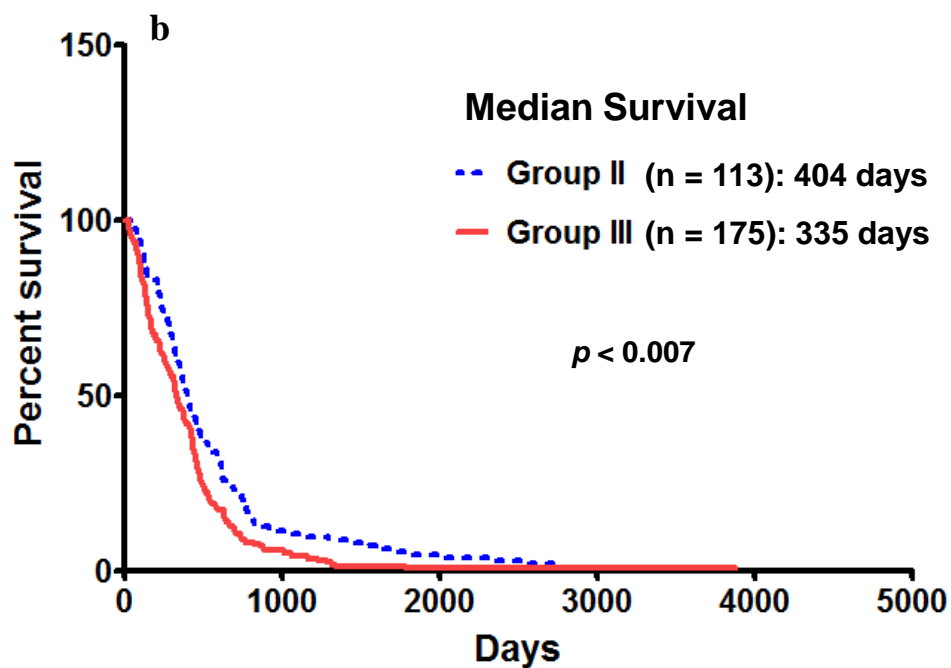

Fig S3

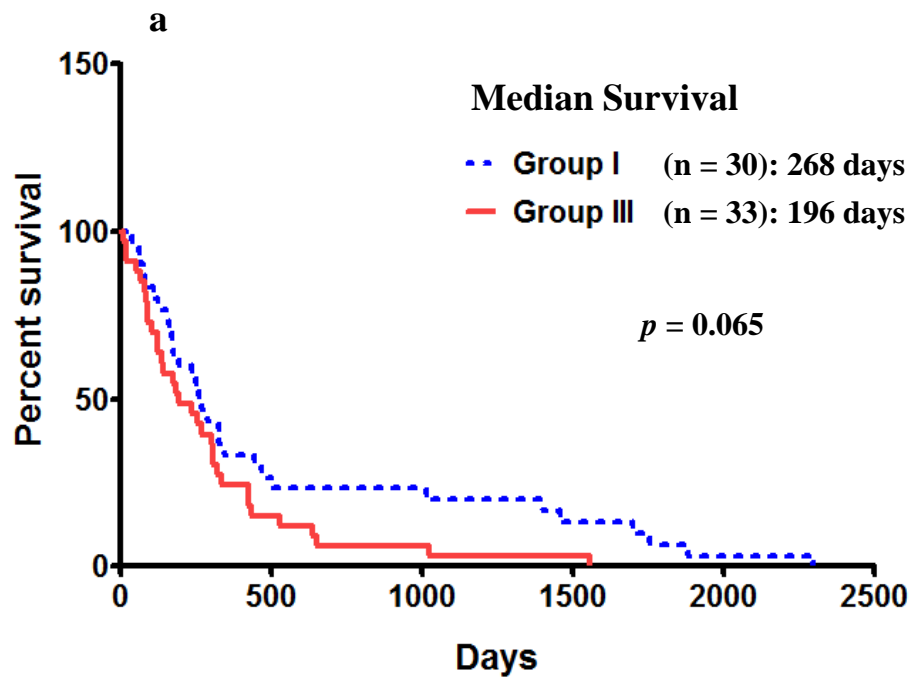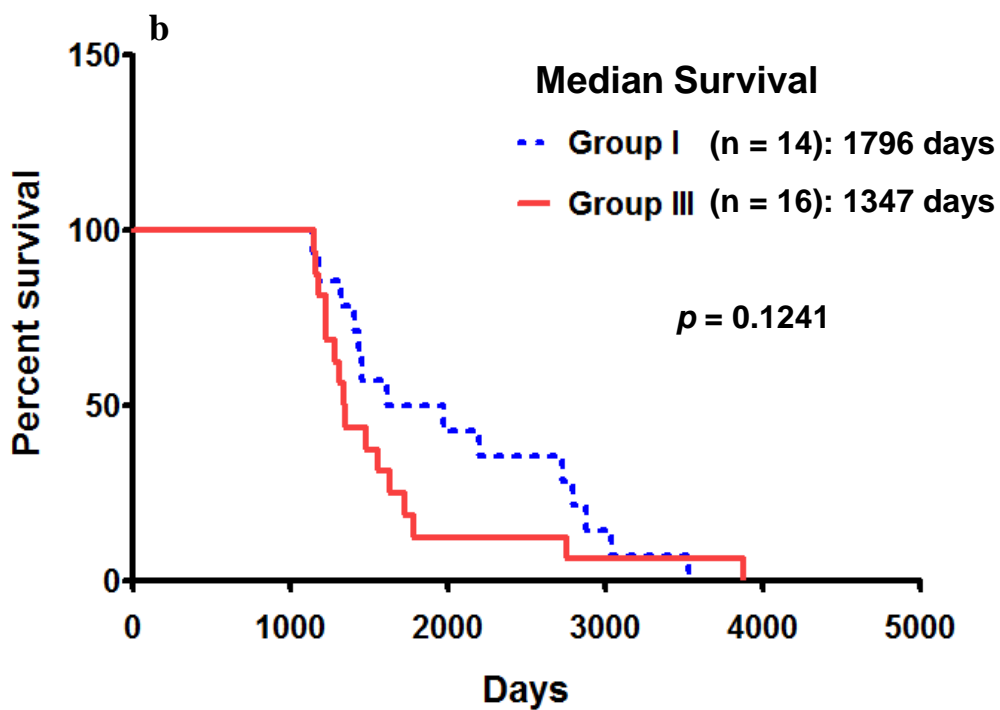

**Fig S4**

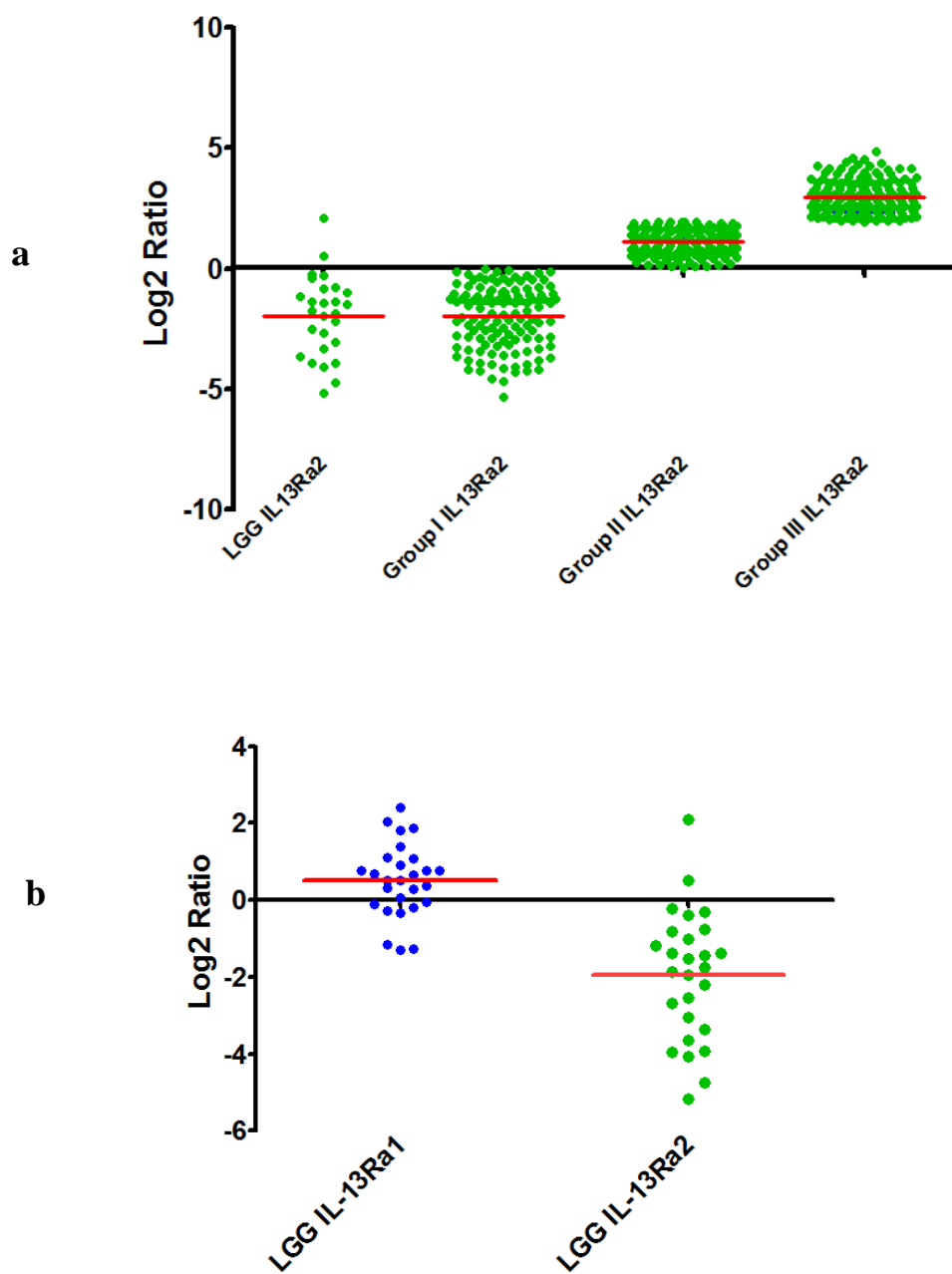

**Fig S5**

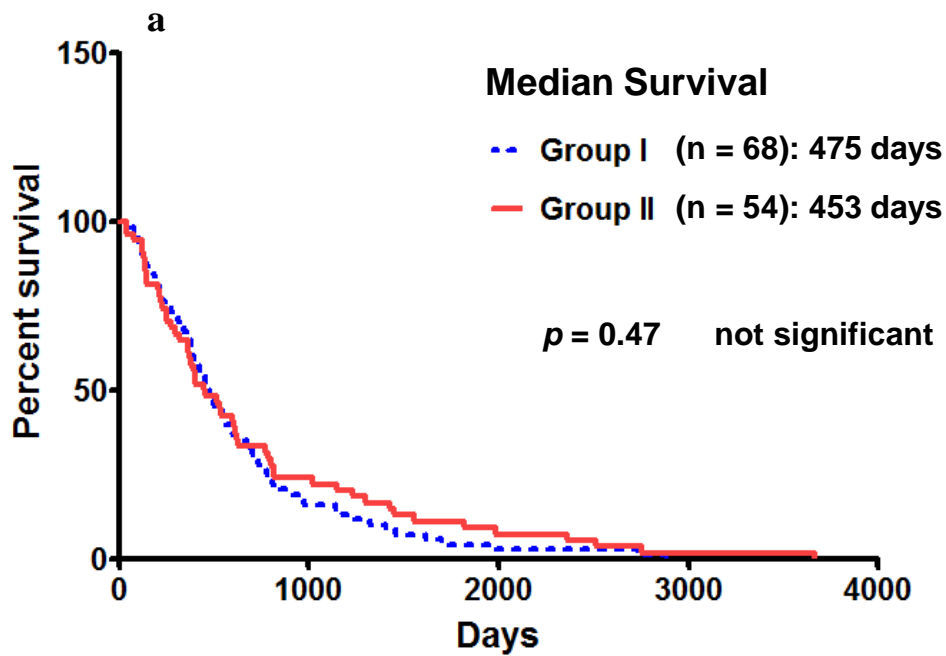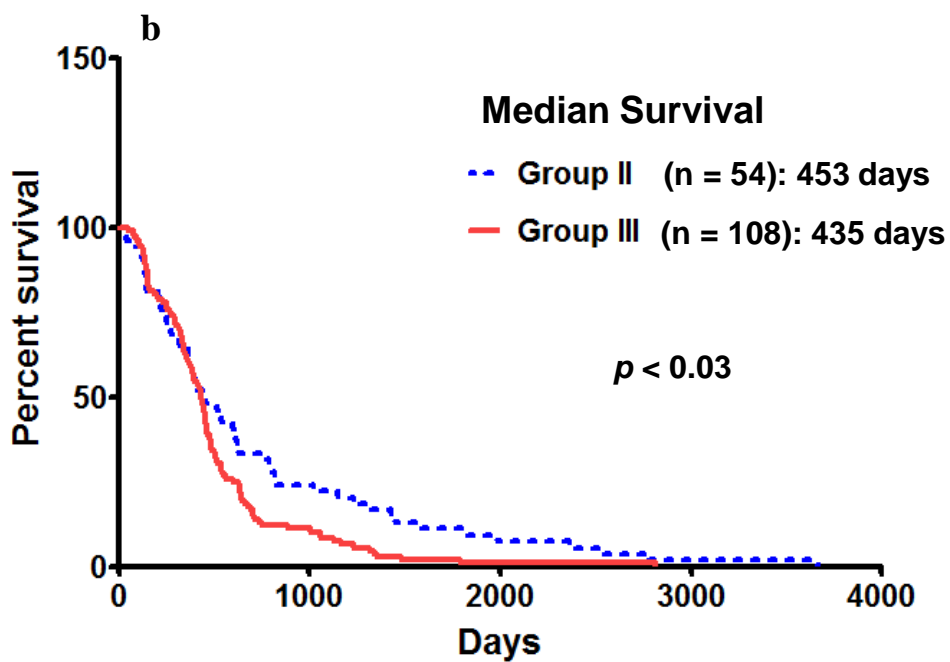

Fig S6

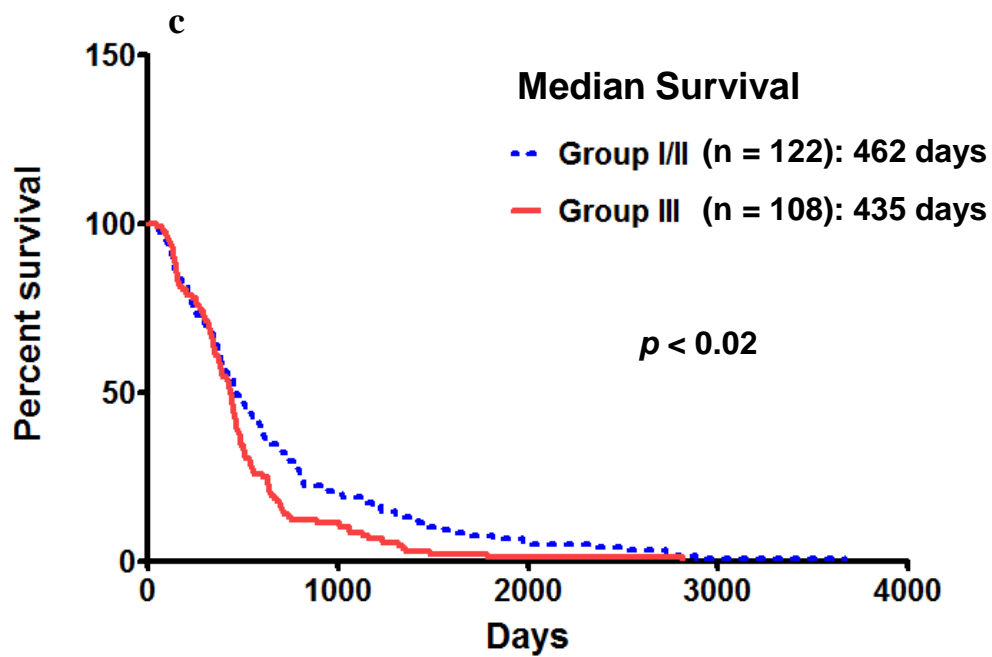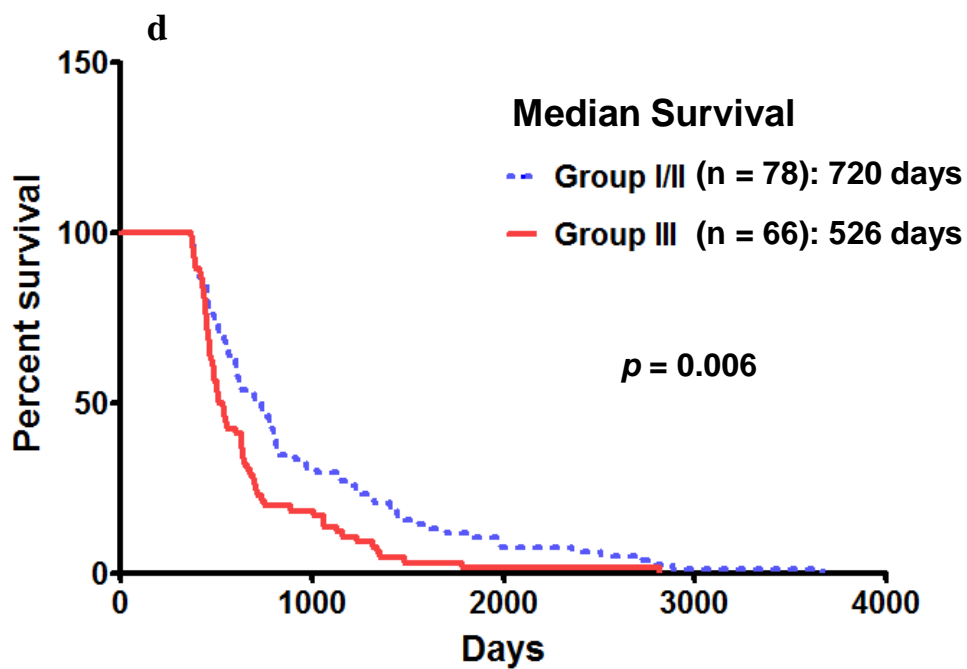

Fig S6

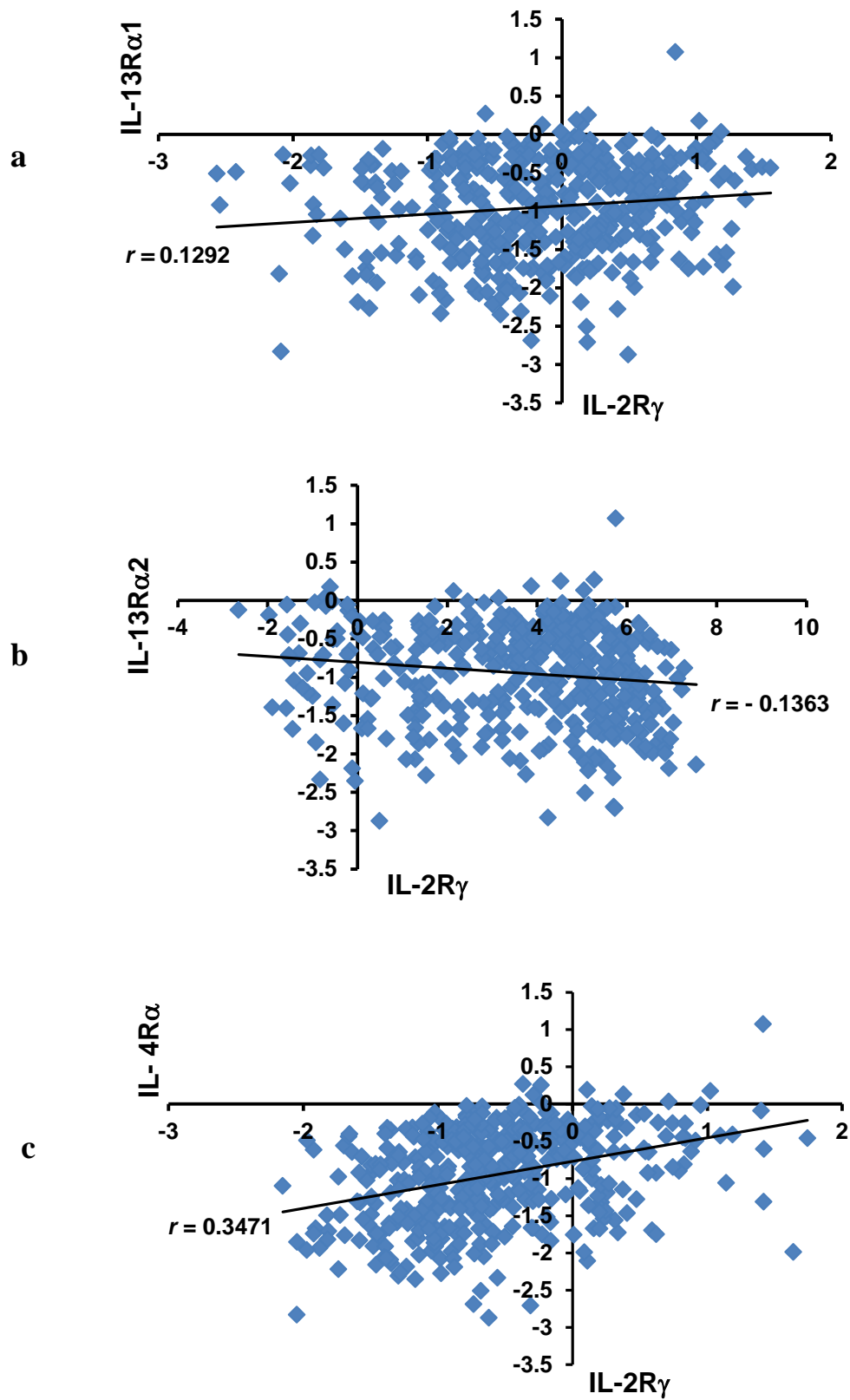

**Fig S7**

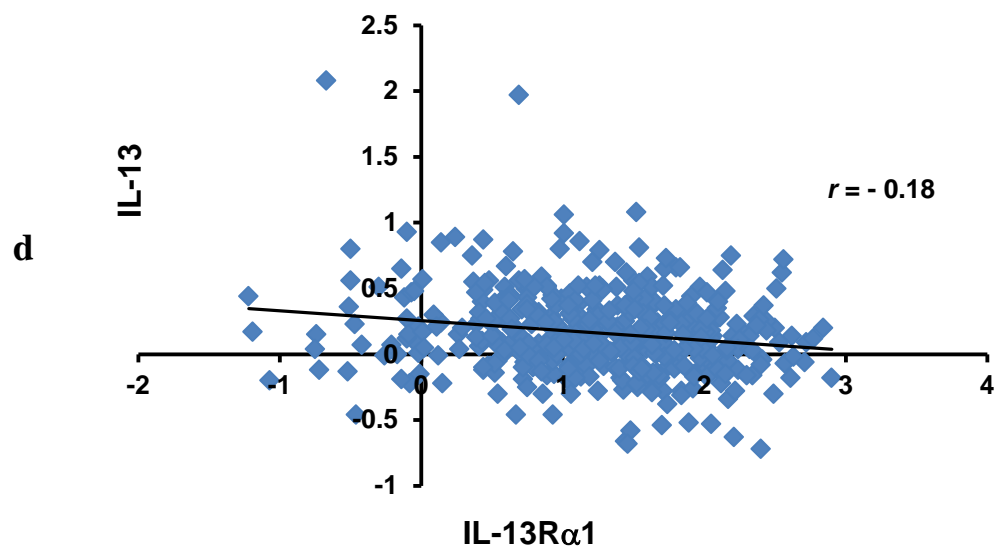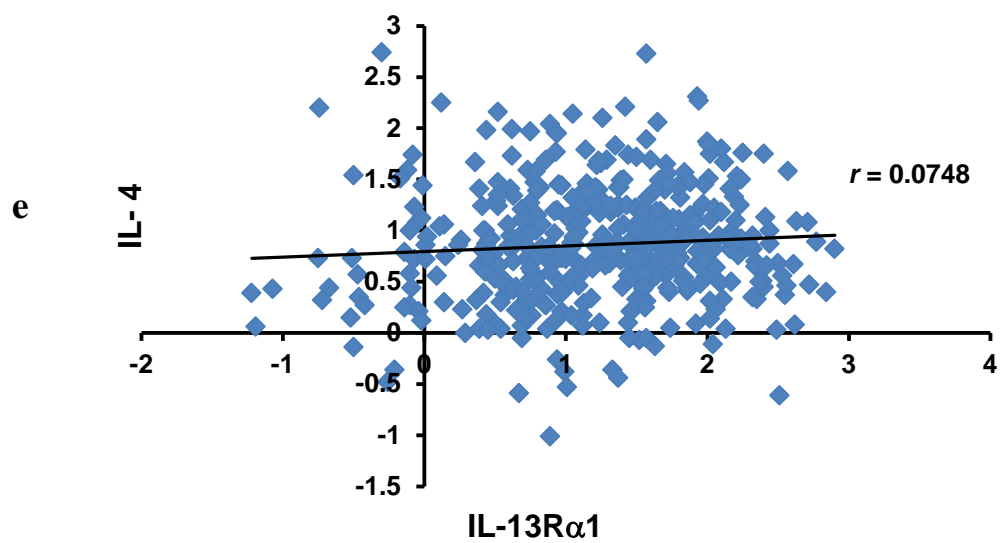

Fig S7

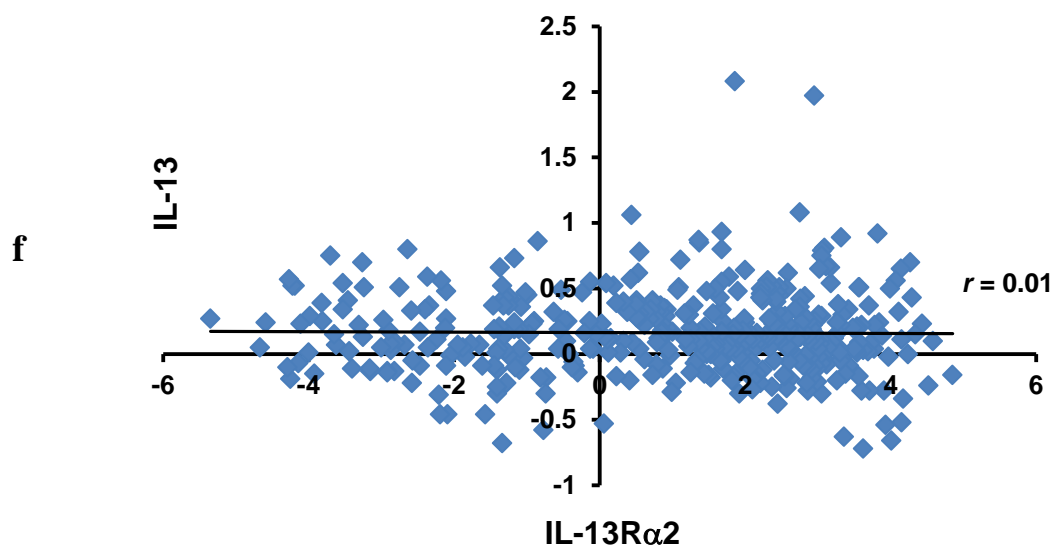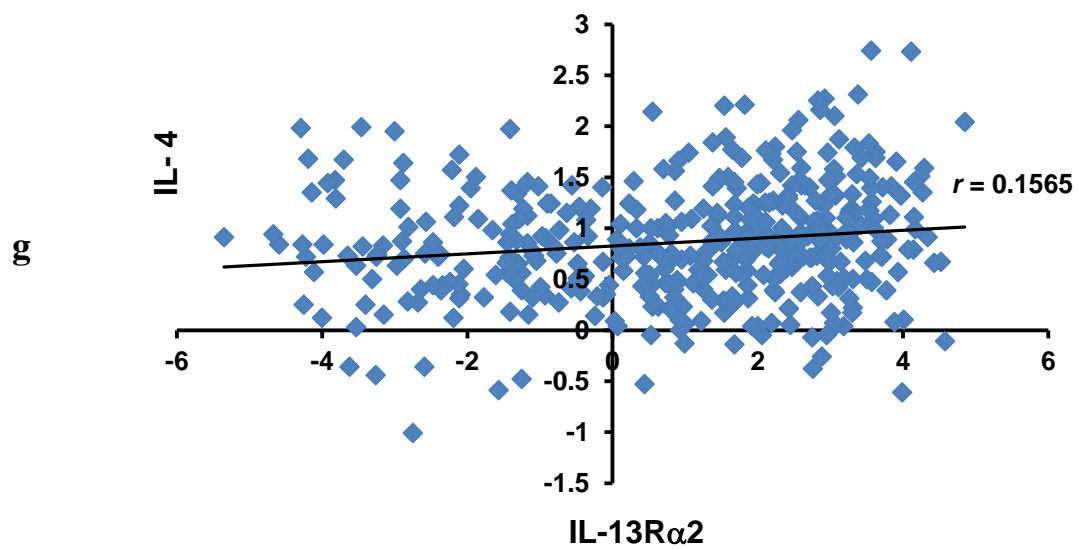

**Fig S7**

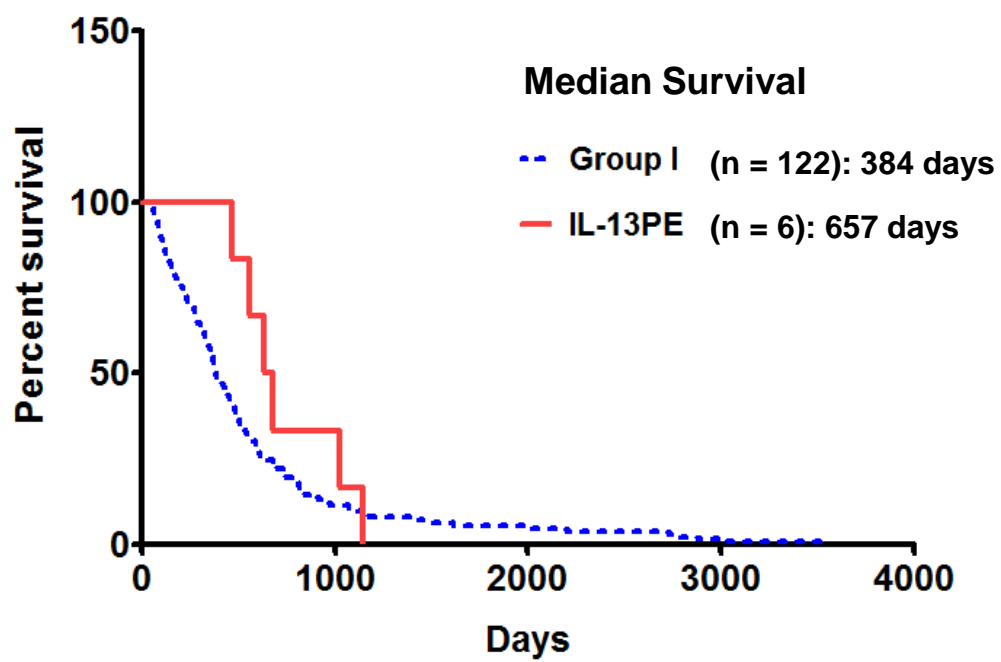

**Fig S8**

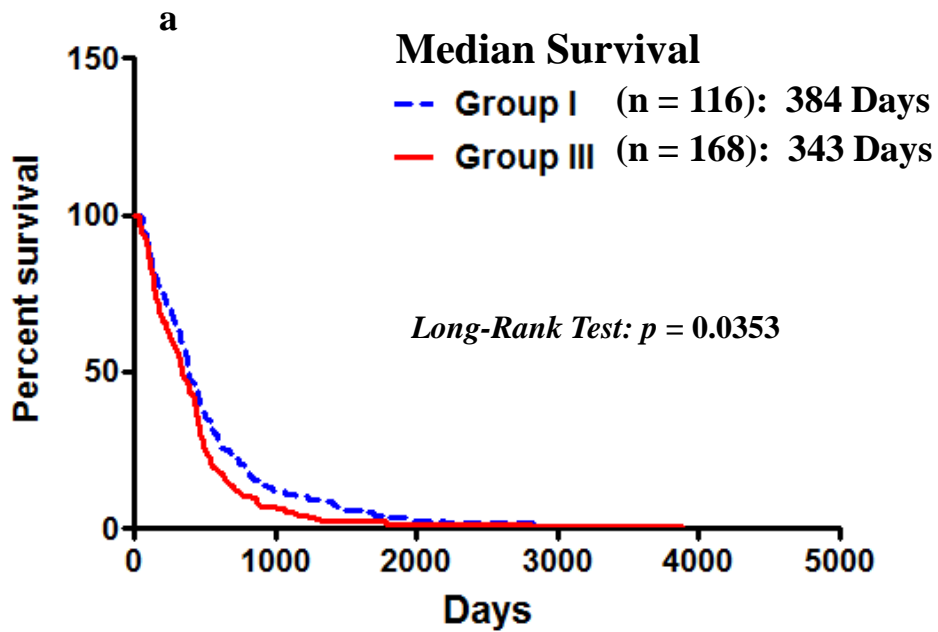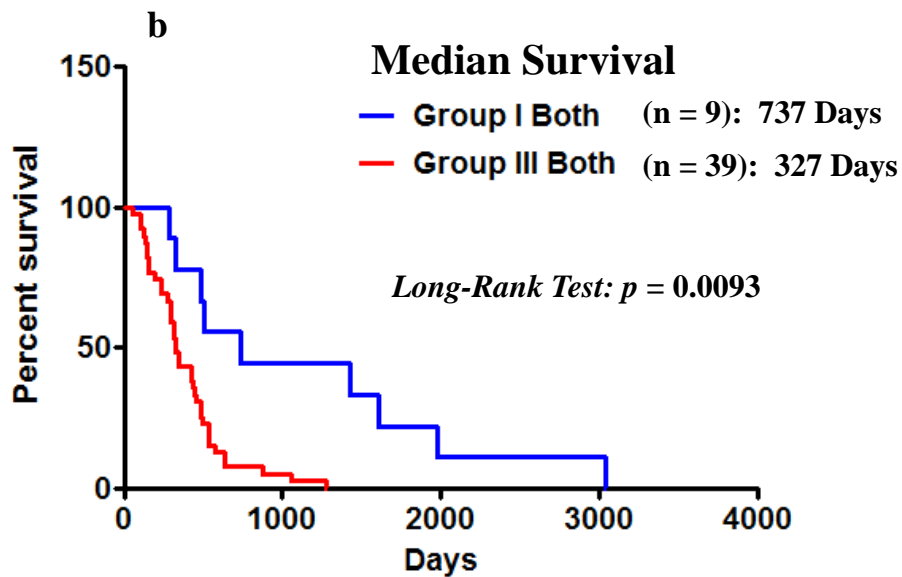

**Fig S9**

Supplement: Supplementary file 2 — Supplementary material 2 (PDF 183 KB) [file 11060_2017_2680_MOESM2_ESM.pdf]
