# Supplementary material for: Analysis of the cancer genome atlas (TCGA) database identifies an inverse relationship between interleukin-13 receptor α1 and α2 gene expression and poor prognosis and drug resistance in subjects with glioblastoma multiforme
Source: J Neurooncol. 2017 Nov 22;136(3):463–74. doi: 10.1007/s11060-017-2680-9 (PMC5805806; doi:10.1007/s11060-017-2680-9)
Supplement: Supplementary file 3 — Supplementary material 3 (DOC 34 KB) [file 11060_2017_2680_MOESM3_ESM.doc]

**Table S2: Correlations between *IL-13Rα2* gene expression and various biomarker genes in GBM**

| **Biomarkers**  **(Gene Symbol)** | ***IL-13Rα2***  **(n = 428)**  ***r* (correlation coefficient)** |
| --- | --- |
| ***CDKN2A*** | 0.041 |
| ***EGFR*** | 0.026 |
| ***IDH1*** | 0.041 |
| ***MGMT*** | 0.001 |
| ***NF1*** | 0.083 |
| ***PDGFRA*** | 0.014 |
| ***TP35*** | 0.134 |

In this analysis, we investigated whether *IL-13Rα2* gene expression is correlated with previously identified gene biomarkers in subgroups of GBM. Correlation between *IL-13Rα2* gene expression and other genes listed in the table was analyzed by Scatter Plot in Excel software, and correlation coefficient was calculated between *IL-13Rα2* gene and each gene. As shown in the table, correlation coefficient between *IL-13Rα2* gene expression and each of the genes listed was very low. No correlation between *IL-13Rα2* gene expression and any of the biomarkers in GBM was identified.
