# Supplementary material for: Analysis of the cancer genome atlas (TCGA) database identifies an inverse relationship between interleukin-13 receptor α1 and α2 gene expression and poor prognosis and drug resistance in subjects with glioblastoma multiforme
Source: J Neurooncol. 2017 Nov 22;136(3):463–74. doi: 10.1007/s11060-017-2680-9 (PMC5805806; doi:10.1007/s11060-017-2680-9)
Supplement: Supplementary file 4 — Supplementary material 4 (PDF 71 KB) [file 11060_2017_2680_MOESM4_ESM.pdf]

Table S1: Immune related genes expressed only in IL-13Rα2 mRNA highly expressed GBM tumors (Group III)

Gene Symbol	Gene Name	Log2 Ratio
**FMOD**	fibromodulin	**1.92**
**CXCL13**	*chemokine (C-X-C motif) ligand 13*	**1.61**
**MNDA**	myeloid cell nuclear differentiation antigen	**1.58**
**HLA-DRA**	major histocompatibility complex, class II, DR alpha	**1.52**
**TLR8**	toll-like receptor 8	**1.50**
**LY75**	lymphocyte antigen 75	**1.48**
**IL15**	interleukin 15	**1.48**
**IL18**	interleukin 18 (interferon-gamma-inducing factor)	**1.45**
**CCR5**	chemokine (C-C motif) receptor 5 (gene/pseudogene)	**1.43**
**FCGR3A**	Fc fragment of IgG, low affinity IIIa, receptor (CD16a)	**1.41**
**PLEK**	pleckstrin	**1.38**
**NOD2**	nucleotide-binding oligomerization domain containing 2	**1.38**
**SLFN11**	schlafen family member 11	**1.37**
**CCL2**	chemokine (C-C motif) ligand 2	**1.37**
**ITGBL1**	integrin, beta-like 1 (with EGF-like repeat domains)	**1.36**
**OSM**	oncostatin M	**1.36**
**WISP1**	WNT1 inducible signaling pathway protein 1	**1.35**
**TREM2**	triggering receptor expressed on myeloid cells 2	**1.35**
**BOC**	Boc homolog (mouse)	**1.33**
**SRGN**	serglycin	**1.33**
**HLA-DRB5**	major histocompatibility complex, class II, DR beta 5	**1.33**
**GJB6**	gap junction protein, beta 6, 30kDa	**1.33**
**HLA-DOA**	major histocompatibility complex, class II, DO alpha	**1.32**
**PARVG**	parvin, gamma	**1.31**
**FCER1G**	Fc fragment of IgE, high affinity I, receptor for gamma polypeptide	**1.27**
**MMRN1**	multimerin 1	**1.25**
**SIGLEC10**	sialic acid binding Ig-like lectin 10	**1.25**
**FYB**	FYN binding protein	**1.25**
**HOXB4**	homeobox B4	**1.24**
**PLA2G4A**	phospholipase A2, group IVA	**1.23**
**IL17B**	interleukin 17B	**1.23**
**TLR6**	toll-like receptor 6	**1.23**
**TLR5**	toll-like receptor 5	**1.23**
**CLEC7A**	C-type lectin domain family 7, member A	**1.22**
**AIF1**	allograft inflammatory factor 1	**1.21**
**HLA-DQA1**	major histocompatibility complex, class II, DQ alpha 1	**1.21**

**PRPH2**	peripherin 2 (retinal degeneration, slow)	**1.21**
**FOXJ1**	forkhead box J1	**1.20**
**TSPO**	translocator protein (18kDa)	**1.20**
**GZMK**	granzyme K (granzyme 3; tryptase II)	**1.20**
**THBD**	thrombomodulin	**1.19**
**SULF1**	sulfatase 1	**1.18**
**ELMOD2**	ELMO/CED-12 domain containing 2	**1.18**
**MFAP4**	microfibrillar-associated protein 4	**1.17**
**IFI6**	interferon, alpha-inducible protein 6	**1.17**
**AIM2**	aminoacyl tRNA synthetase complex-interacting multifunctional protein 2	**1.17**
**DDIT3**	DNA-damage-inducible transcript 3	**1.17**
**BTG3**	B-cell translocation gene 3	**1.16**
**TBX21**	T-box 21	**1.15**
**SERPING1**	serpin peptidase inhibitor, clade G (C1 inhibitor), member 1	**1.15**
**SYK**	spleen tyrosine kinase	**1.14**
**HLA-DQB1**	major histocompatibility complex, class II, DQ beta 1	**1.13**
**VDR**	vitamin D (1,25- dihydroxyvitamin D3) receptor	**1.13**
**CSF3R**	colony stimulating factor 3 receptor (granulocyte)	**1.13**
**C1S**	complement component 1, s subcomponent	**1.13**
**CD1D**	CD1d molecule	**1.12**
**TGFBR1**	transforming growth factor, beta receptor 1	**1.12**
**PSMB10**	proteasome (prosome, macropain) subunit, beta type, 10	**1.12**
**SPI1**	spleen focus forming virus (SFFV) proviral integration oncogene spi1	**1.11**
**SIPA1**	signal-induced proliferation-associated 1	**1.11**
**CIITA**	class II, major histocompatibility complex, transactivator	**1.11**
**LILRB1**	leukocyte immunoglobulin-like receptor, subfamily B 1	**1.11**
**HES1**	hairy and enhancer of split 1, (Drosophila)	**1.11**
**TNFRSF14**	tumor necrosis factor receptor superfamily, member 14	**1.11**
**TIPARP**	TCDD-inducible poly(ADP-ribose) polymerase	**1.10**
**LCP2**	lymphocyte cytosolic protein 2	**1.10**
**FXYD5**	FXYD domain containing ion transport regulator 5	**1.10**
**FPR1**	formyl peptide receptor 1	**1.10**
**CFD**	complement factor D (adipsin)	**1.10**
**ATF5**	activating transcription factor 5	**1.10**
**PSMB8**	proteasome (prosome, macropain) subunit, beta type, 8	**1.10**
**ANXA4**	annexin A14	**1.09**
**IFT20**	intraflagellar transport 20 homolog (Chlamydomonas)	**1.09**
**LCP1**	lymphocyte cytosolic protein 1 (L-plastin)	**1.09**
**CFH**	complement factor H	**1.09**
**IGSF5**	immunoglobulin superfamily, member 5	**1.08**
**SELL**	selectin L	**1.08**

**OXTR**	oxytocin receptor	**1.08**
**IL33**	interleukin 33	**1.08**
**CD226**	CD226 molecule	**1.08**
**F3**	coagulation factor III (thromboplastin, tissue factor)	**1.07**
**CFLAR**	CASP8 and FADD-like apoptosis regulator	**1.07**
**F11R**	F11 receptor	**1.07**
**SLFN13**	schlafen family member 13	**1.07**
**CD84**	CD84 molecule	**1.07**
**TMEM173**	transmembrane protein 173	**1.06**
**HMHA1**	histocompatibility (minor) HA-1	**1.06**
**HSPB1**	heat shock 27kDa protein 1	**1.05**
**TNFAIP3**	tumor necrosis factor, alpha-induced protein 3	**1.05**
**A2M**	alpha-2-macroglobulin	**1.05**
**CD37**	CD37 molecule	**1.04**
**LAMP2**	lysosomal-associated membrane protein 2	**1.04**
**NRP2**	neuropilin 2	**1.03**
**IL7R**	interleukin 7 receptor	**1.03**
**AMICA1**	adhesion molecule, interacts with CXADR antigen 1	**1.03**
**NRP1**	neuropilin 1	**1.03**
**BCAP29**	B-cell receptor-associated protein 29	**1.03**
**IFI35**	interferon-induced protein 35	**1.02**
**FLI1**	Friend leukemia virus integration 1	**1.02**
**MAGED1**	melanoma antigen family D, 1	**1.02**
**IFT52**	intraflagellar transport 52 homolog (Chlamydomonas)	**1.02**
**CD276**	CD276 molecule	**1.02**
**TBXAS1**	thromboxane A synthase 1 (platelet)	**1.02**
**RRAD**	Ras-related associated with diabetes	**1.01**
**CXCR4**	chemokine (C-X-C motif) receptor 4	**1.01**
**CD86**	CD86 molecule	**1.01**
**EMILIN1**	elastin microfibril interfacer 1	**1.00**
**FCER1A**	Fc fragment of IgE, high affinity I, receptor for alpha polypeptide	**1.00**
**IL20RB**	interleukin 20 receptor beta	**1.00**
**IRF7**	interferon regulatory factor 7	**0.99**
**TNFRSF1B**	tumor necrosis factor receptor superfamily, member 1B	**0.99**
**PLCG2**	phospholipase C, gamma 2 (phosphatidylinositol-specific)	**0.98**
**IL4**	interleukin 4	**0.98**
**CFB**	complement factor B	**0.98**
**TBX5**	T-box 5	**0.98**
**FCN3**	ficolin (collagen/fibrinogen domain containing) 3	**0.97**
**PRAM1**	PML-RARA regulated adaptor molecule 1	**0.97**
**IL18RAP**	interleukin 18 receptor accessory protein	**0.97**
**CPA3**	carboxypeptidase A3 (mast cell)	**0.96**

**SHFM1**	split hand/foot malformation (ectrodactyly) type 1	**0.96**
**DPP4**	dipeptidyl-peptidase 4	**0.96**
**SERPINI2**	serpin peptidase inhibitor, clade I (pancpin), member 2	**0.96**
**RHCE**	Rh blood group, CcEe antigens	**0.96**
**KCNJ8**	potassium inwardly-rectifying channel, subfamily J, member 8	**0.96**
**COL9A1**	collagen, type IX, alpha 1	**0.95**
**CD164**	CD164 molecule, sialomucin	**0.95**
**DUSP6**	dual specificity phosphatase 6	**0.95**
**IFNAR1**	interferon (alpha, beta and omega) receptor 1	**0.95**
**IRAK3**	interleukin-1 receptor-associated kinase 3	**0.95**
**LST1**	leukocyte specific transcript 1	**0.95**
**ICAM1**	intercellular adhesion molecule 1	**0.95**

*Log2 ratios are the mean values of immune related genes expressed in IL-13Rα2 highly expressed tumor group (Group III).
